# Longitudinal validity and reliability of the Myeloma Patient Outcome Scale (MyPOS) was established using traditional, generalizability and Rasch psychometric methods

**DOI:** 10.1007/s11136-017-1660-z

**Published:** 2017-07-27

**Authors:** Christina Ramsenthaler, Wei Gao, Richard J. Siegert, Steve A. Schey, Poly M. Edmonds, Irene J. Higginson

**Affiliations:** 1 Department of Palliative Care, Policy and Rehabilitation, School of Medicine, Cicely Saunders Institute, King’s College London, London, SE5 9PJ UK; 2 Auckland University of Technology, Auckland, New Zealand; 3 Department of Haematological Medicine, King’s College Hospital NHS Foundation Trust, London, UK; 4 Department of Palliative Care, King’s College Hospital NHS Foundation Trust, London, UK

**Keywords:** Multiple myeloma, Health status, Rasch analysis, Quality of life, Responsiveness

## Abstract

**Purpose:**

The Myeloma Patient Outcome Scale (MyPOS) was developed to measure quality of life in routine clinical care. The aim of this study was to determine its longitudinal validity, reliability, responsiveness to change and its acceptability.

**Methods:**

This 14-centre study recruited patients with multiple myeloma. At baseline and then every two months for 5 assessments, patients completed the MyPOS. Psychometric properties evaluated were as follows: (a) confirmatory factor analysis and scaling assumptions (b) reliability: Generalizability theory and Rasch analysis, (c) responsiveness and minimally important difference (MID) relating changes in scores between baseline and subsequent assessments to an external criterion, (d) determining the acceptability of self-monitoring.

**Results:**

238 patients with multiple myeloma were recruited. Confirmatory factor analysis found three subscales; criteria for scaling assumptions were satisfied except for gastrointestinal items and the *Healthcare support* scale. Rasch analysis identified limitations of suboptimal scale-to-sample targeting, resulting in floor effects. Test–retest reliability indices were good (*R* = > 0.97). Responsiveness analysis yielded an MID of +2.5 for improvement and -4.5 for deterioration.

**Conclusions:**

The MyPOS demonstrated good longitudinal measurement properties, with potential areas for revision being the Healthcare Support subscale and the rating scale. The new psychometric approaches should be used for testing validity of monitoring in clinical settings.

**Electronic supplementary material:**

The online version of this article (doi:10.1007/s11136-017-1660-z) contains supplementary material, which is available to authorized users.

## Introduction

Cancer is a major public health concern, being the second leading cause of death worldwide [1]. With the ageing of the society, cancer incidence is rising [2, 3]. Despite advances in treatments, many cancer patients still face long disease trajectories and incurable disease. Multiple myeloma, an incurable cancer of the bone marrow and the second most common haematological malignancy [4], exemplifies this changing face of cancer. Many myeloma patients experience a more chronic disease trajectory, coping with gradually progressing disease, interspersed with intervals of stable disease with minimal or maintenance treatment but lasting effects of high-dose chemotherapy [5, 6]. This longer disease trajectory of cancer and the intensive treatments have led to a need to evaluate patient-reported outcomes in addition to traditional monitoring, such as response to treatment and toxicity profiles, in this condition.

Patient-reported outcomes primarily comprise symptoms and health-related quality of life (HRQOL). Incorporating longitudinal assessment into routine clinical practice has shown benefits such as better symptom control, improved patient-clinician communication and satisfaction with care [7, 8]. In trials, serial assessment of HRQOL incorporates the patient’s experience while monitoring treatment safety and efficacy [9]. It also aids prognosis in chronic conditions and in haematological malignancy [10–12].

Despite these benefits, few measures are designed for monitoring HRQOL in routine clinical settings [13, 14]. A systematic review of 13 generic and disease-specific HRQOL measures in multiple myeloma [13] found no single tool developed or validated for this purpose. Consequently, the Myeloma Patient Outcome Scale (MyPOS), a questionnaire to measure disease-specific HRQOL and palliative care concerns, was developed and validated in a cross-sectional sample of 380 community and inpatient myeloma patients in the United Kingdom (UK) [15]. However, the clinical utility of the MyPOS in form of longitudinal validity and reliability [16–19] still needs to be established.

The psychometric criteria for longitudinal monitoring validity are still ill-defined. Traditional psychometrics and associated guidelines focus on usages of assessment or screening [20–22]. The notable exception is McHorney’s study of individual patient-monitoring in which the following criteria were proposed [23]: (i) practical features (brief measures, easy administration, easy score interpretation), (ii) breadth of health measured (variety of health concepts with assessing the full range of health from disability to well-being), (iii) depth of health measured (minimal floor and ceiling effects), (iv) precision for cross-sectional assessment (precise reliability estimates, e.g. Cronbach’s alpha, with small standard error of measurement) (v) precision for longitudinal monitoring (high reproducibility/test–retest reliability with small standard error of measurement), and (vi) validity (satisfactory convergent/divergent validity, high responsiveness/sensitivity to clinical change and definition of individual patient application, e.g. screening, monitoring, decision-making, tested). The authors also recommend more stringent benchmarks for measurement errors to fit the longitudinal use of measures [23]. Building on this work, we propose to extend McHorney et al’s framework by incorporating new psychometric approaches, particularly Rasch analysis [24, 25] and Generalizability theory [26–28], to further test some of their six quality criteria for longitudinal monitoring applications. Particularly Generalizability theory has been used successfully in psychological studies that monitored emotional changes [29]. Both techniques are suitable since they address the limitations of classical test theory (CTT) by providing individual item information, information on item invariance and person-level indicators that help understand floor and ceiling effects, understanding sources of measurement error, and the ability for discriminating among different patient groups (i.e. disease severity) [24, 25, 28, 30]. In particular, we propose to extend analysis for criteria (iii), depth of health measured, (iv) precision for cross-sectional assessment, and (v) precision for longitudinal monitoring by using person-item targeting in Rasch analysis to further understand floor and ceiling effects, and to use the variance decomposition method for forming reliability indices beyond simple test–retest reliability, to understand how reliable the use of an instrument is in the situation of screening HRQOL at one point in time, monitoring HRQOL over time and detecting change over time (iv and v, [29]).

We aim to examine the longitudinal validity and reliability of the MyPOS. The objectives are: (a) to evaluate the validity of the MyPOS and its scale in myeloma patients at different stages in their disease trajectory, (b) to determine the reliability of the MyPOS over time (test–retest reliability) within a Generalizability framework, (c) to determine the responsiveness and clinical significance of changes in quality of life scores and subscale scores and estimate the minimal important change (MID), both for patients who deteriorated and improved, and (d) to explore the acceptability of frequent self-monitoring of HRQOL.

## Methods

### Study design and participants

This multi-centre, prospective longitudinal study recruited patients with multiple myeloma at different disease stages. Patients were enrolled in the study from March 2014 until July 2015. Inclusion criteria were as follows: older than 18 years, confirmed diagnosis of multiple myeloma that had been disclosed to the patient and capacity to give informed written consent. Patients who were too unwell, distressed or symptomatic to participate, as judged by their clinical team, were excluded, as were patients with severe neutropenia or for whom myeloma was not the most important health problem. Patients were recruited from 14 hospital trusts in the United Kingdom, both from secondary and tertiary centres. Study procedures followed the guidelines of the Helsinki Declaration. Ethical and research governance approvals were obtained from the Central London Ethics Committee (13/LO/1140) with further local Research and Development approvals from all participating National Health Service (NHS) hospital trusts.

### Procedures

Consenting patients were invited to complete questionnaires at baseline and then every two months for a total of five assessments and a maximum follow-up time of eight months post-baseline. The first questionnaire was given to patients when they attended outpatient clinics. Subsequent questionnaires were sent via mail, with a self-addressed, pre-stamped envelope provided for return, a pen and a sweet to boost participation [31]. Patients were followed, if possible, if they moved to a nursing home, hospital or hospice. We sought information about any deaths that occurred.

### Questionnaires

Participants completed the MyPOS [15]. The MyPOS is a module of the Palliative Care Outcome Scale (POS) [32–34], extended by myeloma-specific concerns. It comprises a list of 13 symptoms and 20 items about quality of life or palliative care concerns. The POS is a prominent measure of palliative care concerns. During the development phase of the MyPOS, in focus groups with experts as well as in cognitive interviews with patients, it was opted to adapt an existing questionnaire rather than develop a new one [35]. In the cognitive interviews, a clear preference for the item style and response options of the POS was shown. Also, some of the generic POS items were used in building the MyPOS since they measured relevant domains of myeloma-related quality of life [35]. In an attempt to harmonise disease- or condition-specific measures of the POS, the Integrated Palliative care Outcome Scale (IPOS) [36] was formed and it was opted by the POS research group to convert all disease-specific POS measures to a common, module-style format (similar to the European Organization for the Research and Therapy of Cancer (EORTC) quality of life and the Functional Assessment of Cancer Therapy (FACT) quality of life questionnaires [37, 38]). At the same time, the POS was revised and especially its original two symptom items were extended by a list of symptoms prevalent in palliative care patients. The revised IPOS now contains 17 items. It is a valid and reliable measure [36]. Just prior to commencing this longitudinal validation study, the MyPOS was converted to become a module of the IPOS. All symptom (generic and myeloma-specific) and general palliative care-related problem items (list extended by four general palliative care-related concerns) now form the first part of the MyPOS and the myeloma-specific concerns form the third part of the questionnaire (for original and revised version see Supplemental Figs. 1, 2). The MyPOS used in this study therefore contains six additional IPOS items not contained in the version validated in Osborne et al. (2015) [15].

Items are scored on a five-point Likert scale. For symptom items, the scale ranges from 0 ‘not at all’ to 4 ‘overwhelmingly’. For all other items, response options labels are question-specific with 0 signifying no problems and 4 signifying a high amount of problems (Supplemental Fig. 3 shows the response options for each scale of the MyPOS). Content and construct validity of the original MyPOS have been established in a clinically representative sample [15, 35].

To evaluate the responsiveness and minimal important change on the MyPOS, an independent question to assess the degree of change was used. This global rating of change question (GRC) [22, 39] asked ‘Has your overall quality of life changed since the first time you completed this questionnaire?’, with patients indicating whether their quality of life had got worse, stayed the same, or had improved. The GRC question was part of each follow-up assessment.

The questionnaire sent at the third assessment contained three open-ended questions to explore the acceptability of frequent self-monitoring. The questions concerned the suitability of the MyPOS for monitoring quality of life, the potential usefulness of monitoring quality of life and how results could be used by patients and clinicians.

### Statistical analysis

Table 1 provides an overview of analyses methods per objective, following the McHorney et al. framework [23], and detailing the criteria that were used for establishing fit and validity/reliability. All quantitative data analyses were conducted in SPSS v.22 [40], lavaan package in R [41] and partial credit Rasch models were run in RUMM2030 [42]. Patients with three or more missing MyPOS questionnaires at the follow-up time points were excluded from statistical analyses. If more than 50% of responses within a scale were missing from one questionnaire, it was removed from the analysis. Missing data in the confirmatory factor analysis were imputed using a multiple imputation approach [43]. Responsiveness analyses and Rasch analysis used a complete case analysis without imputation of missing data.

**Table 1:** Overview of measurement properties and criteria for assessing longitudinal validity and reliability

Measurement property	Statistical methods
*Objective 1: Further validity of the MyPOS*	
Diagonally weighted least squares (DWLS) confirmatory factor analysis using R lavaan package [41]	Goodness-of-fit indices: (a) *χ* ^2^/df > 2 [44] (b) Comparative fit index (CFI) of ≥ 0.90 [45] (c) Root mean square error of approximation (RMSEA) of ≤ 0.06, 90% confidence interval 0.05–0.08 [45] (d) Non-normal fit index (NNFI) of ≥ 0.95 or normal fit index (NFI) of ≥ 0.95 [45] Checks of unidimensionality of three separate subscales analysed with Rasch analysis: principal component analysis and paired t-tests in RUMM2030 [46, 47]: (a) RMSEA < 0.08 [48] (b) CFI > 0.90 [49] (c) Tucker-Lewis Index (TLI) > 0.90 [45]
Floor and ceiling effects via descriptive and Rasch analysis	Data completeness and distribution of item responses >15% of responders at the lower or upper end of the scale [16] Rasch analysis: Scale-to-person targeting, the ability of the scale to cover the whole range of person estimates, shown on the person-item threshold distribution map [29]
Scaling assumptions via Rasch analysis (RUMM 2030) [42]	
Fit to the Rasch model	*Fit to the Rasch model* Non-significant *Χ* ^*2*^-test [50] and RMSEA < 0.2 [45]. However, large sample size can inflate the *Χ* ^*2*^ value and increase the likelihood of identifying misfit [45]. A partial credit Rasch model was used
Fit of individual items	*Individual item fit* Fit residual range −2.5 to +2.5 [50]. The residuals indicate the level of agreement between the observed and expected responses with perfect fit being given if a mean residual is zero with a standard deviation falling between −1 and +1. Positive fit indices above +2.5 show misfit to the model, negative fit indices below −2.5 indicate redundancy of items. Item characteristic curves were examined for graphical item fit
Person fit	*Person fit* Same criteria as item fit
Reliability	*Reliability*: Person Separation Index (measure of internal consistency in Rasch analysis) ≥ 0.70 [51]
Response options	*Response options:* Category probability curve maps for each item examined for disordered answer options, signifying ambiguous labelling or abundance of response options
Redundant items	*Redundant items* Residual correlation matrix, identifying pairs of items with high correlation coefficients (≥0.3) [50]
*Objective 2: Test*–*retest reliability/item invariance of the MyPOS*	
Test–retest reliability using Generalizability theory	Restricted maximum-likelihood variance decomposition (VARCOMP) with participants and interaction terms as random factors and items and days as fixed factors. The variance associated with each component of variation, systematic between-person differences in mean item levels, true within-person change over time, idiosyncratic item responses and random measurement error, is partitioned [27, 28]. These variance estimates are used to form indices of the reliability for discriminating between-persons (between-person differences) and within-person change Four generalizability coefficients (all >0.5 [29]): *R*_*1F*_ Reliability of assessment/screening (Is the MyPOS reliable at each assessment?) *R*_*1R*_ Reliability of discrimination (Can the MyPOS reliably distinguish between persons over time?) *R*_*KF*_ Test–retest reliability (Is the MyPOS reliable over time?) *R*_*c*_ Within-person reliability of change (Can the MyPOS assess change in one person over time?) It should be noted that determination of test–retest reliability within Generalizability theory is a model-based approach that derives reliability indices from variance decomposition as an alternative way to intra-class correlation coefficients. Analysis of test–retest reliability was based on the subgroup of stable patients as indicated by the global rating of change (“unchanged”—see objective 3, responsiveness)
Item invariance using Rasch analysis	Differential item functioning (DIF) via a two-way ANOVA of standardised residuals with Bonferroni correction for type I error [52]; assessing whether item mean scores showed significant change over all five assessments [50] Significant interaction between class interval (level of quality of life) and time indicates a non-uniform DIF and an unstable, unreliable item
*Objective 3: Responsiveness and minimal important difference (MID(for MyPOS*	
Responsiveness	GRC to categorise patients into: (a) improved overall QOL (b) deteriorated overall QOL (c) unchanged Differences in mean score changes between each time point and baseline were assessed and graphed. The adequacy of the anchor was assessed via Spearman correlation [17]
MID: anchor-based approach	Receiver-operating characteristic curve (ROC) to determine optimal cut-off points separately for improvement and deterioration, according to GRC ratings [53]. MID: cut-off point on ROC for which the sum of percentages of false-positive and false-negative classifications [(1-sensitivity or true positive rate) + (1-specificity or false positive rate)] is smallest [39]. Significance of the area under the curve with a *p* value > 0.5 indicates changes on the MyPOS scores are associated with the gold standard GRC criterion [39]. Graph of distribution of change scores, MIDs and 95% CIs [54]
MID: distribution-based approach	Standard deviation at baseline used to estimate MID [55] Following Cohen’s criteria [56], small changes (0.2 × SD), moderate changes (0.5 × SD) and large changes (0.8 × SD) were computed. A moderate effect size change was designated as the MID [55]
*Objective 4: acceptability of monitoring*	
Acceptability	Thematic analysis of responses to open-ended questions about views on self-monitoring and data feedback were analysed using thematic analysis [57]

For construct validity (*objective 1*), re-evaluating the subscale structure defined in the initial validation [15] was necessary due to the sample-dependency of CTT approaches [58]. Confirmatory factor analyses contrasting three models to find best fit of the data were used: (i) a uni-dimensional model (one factor) solution, (ii) three-factor solution replicating the solution from the initial validation [15] with symptom and functioning items loading on one factor, separate from factors emotional response and healthcare support, and (iii) an adapted three-factor solution with all functioning items loading onto the emotional response factor, resulting in three subscales Symptoms, Functioning and Emotional response, and Healthcare support. Scaling assumptions of the total MyPOS score, subscale scores and individual item scores were evaluated using Rasch analysis. A partial credit Rasch model was fitted to each subscale, *Symptoms* (13 items), *Emotions* (17 items) and *Healthcare Support* (3 items), separately. Floor/ceiling effects and distribution of item responses were checked using descriptive statistics and Rasch analysis (person-location maps). The presence of floor or ceiling effects is indicated in the person-location map by mean item location scores not matching the whole range of person locations at the lower or upper end of the scale [59]. This indicates either items missing from the measure to represent very good or poor HRQOL, or indicates that the sample used for evaluation of the measure is not well targeted to comprise all levels of severity that the MyPOS measures [50]. For establishing the test–retest reliability and invariance of the MyPOS (*objective 2*) for participants that indicated they did not experience a change in their condition over time, the Generalizability theory framework was used [26–28]. Four generalizability coefficients [29] were computed (see Table 1). Item invariance was further tested using Rasch analysis following Hobart et al.*’s* [58] approach using differential item functioning (DIF). DIF is an indicator of items not performing in a stable/invariant way since the expected values on the item are not the same for all subgroups in the sample (i.e. groups of different disease severity or functional ability) [58]. *Objective 3*, establishing the responsiveness to change and the minimal important difference for the MyPOS, followed guidelines by Guyatt [55] and used a combination of anchor-based, distribution-based approaches. For responsiveness, we used the GRC to identify patients that experienced change over time, with categories improved, unchanged and deteriorated to examine the differences in mean score changes between each time point and baseline (T2 to T1, T3 to T1, T4 to T1, T5 to T1). We determined ROC curves separately for improvement and deterioration (improved vs. stable or deteriorated vs. stable) for total MyPOS score and the three subscale scores. For *objective 4*, we analysed participants’ written comments in the open-ended questions of the MyPOS using thematic analysis [57].

## Results

### Characteristics of patients and questionnaire completion

250 patients were recruited of whom 238 completed the questionnaire at baseline. Mean age was 68.5 (range 34–92 years), mean time post diagnosis was 3.3 years with 139 (25.5%) patients who had been living with myeloma 5 years and longer (see Table 2). 199 participants completed time point 2 (83.6%), 171 completed time point 3 (71.8%), 150 completed time point 4 (63%) and 125 (52.5%) completed the last time point 5 questionnaire. Of the 113 patients lost to follow up, 9 had died, 17 had been feeling too unwell to continue with the study, 2 had moved, and 86 gave no reason for discontinuing the study. 12 questionnaires were lost in the mail.

**Table 2:** Demographic and clinical characteristics of 238 patients with myeloma included in the study

Variable	
Age, mean ± SD (range)	68.5 ± 10.5 (range 34–92)
Men, *N* (%)	147 (61.8)
Married, *N*(%)	170 (71.4%)
White background, *N*(%)	220 (92.4%)
Education level, *N*(%)	
Secondary school	137 (57.5)
Technical qualification	52 (21.8)
University	41 (17.3)
Working, *N*(%)	41 (17.2)
Type of myeloma, *N*(%)	
IgA or IgG	180 (78.6)
Light chain disease	39 (16.4)
Other	9 (3.8)
ISS stage at diagnosis, *N*(%)	
I	68 (28.6)
II	41 (17.2)
III	52 (18.6)
Time since diagnosis (in months), mean (SD)	39.1 (38.2)
Disease stage, *N*(%)	
Newly diagnosed	38 (15.9)
Stable/plateau	128 (53.8)
Relapsed/progressive/refractory disease	72 (30.3)
Currently receiving treatment, *N*(%) 118 (49.6)	
Active therapy	80 (33.6)
Maintenance therapy	38 (15.9)
Intensity of treatments received, *N*(%)	
Chemotherapy only	111 (46.7)
Chemotherapy and HSCT	76 (31.9)
Two or more HSCT	15 (6.3)
Lines of treatment received, mean (SD)	1.5 (1.2)
ECOG performance status, *N*(%)	
0 Fully active	79 (33.2)
1 Restricted	104 (43.7)
2 Unable to work	33 (13.9)
3 or 4—Limited self-care/bed-bound	15 (6.3)
Charlson comorbidity index, mean (SD)	4.9 (1.5)
General symptom level (MyPOS), *N*(%)	
0	3 (1.3)
1–5	70 (29.4)
6–8	65 (72.3)
9–15	92 (38.7)
Mean number of symptoms, Mean ± SD	7.4 ± 3.6
Total MyPOS, mean ± SD	26.0 ± 16.8

Initial induction and HSCT were counted as one single line of treatment. Likewise, if during a line of treatment the anti-myeloma therapy was changed due to unresponsiveness or side effects, this was still counted as one line. If active treatment was followed by maintenance treatment, active and maintenance were counted as one line. A treatment-free interval was defined by not receiving active or maintenance anti-myeloma therapy, whereas supportive therapies (e.g. bisphosphonates or anti-anaemia treatment) were possible

*ECOG* Eastern Cooperative Oncology Group performance status; *HSCT* haematopoietic stem cell transplantation; *IgA* immunoglobulin A; *IgG* immunoglobulin G; *ISS* international staging system for multiple myeloma; *MyPOS* Myeloma Patient Outcome Scale; *SD* standard deviation

At baseline, 3.3% of responses in returned questionnaires were missing. The number of missing responses reduced over time: 1.2% at time point 2, 0.7% at time point 3, 0.7% at time point 4 and 0.9% at last follow-up time point.

### Confirmatory factor analysis of the MyPOS and Rasch scaling

#### Confirmatory factor analysis

Factor analysis confirmed a three-factor structure but with functioning items now loading onto the Emotional response factor (solution iii). The fit indices indicated a satisfactory model fit. Although the *X* ^*2*^ test was significant, the RMSEA (0.056; 90% CI 0.050–0.063) and the CFI (0.942) were satisfactory. When compared to the uni-dimensional solution, the three-factor solution performed best. The three factors together explain a total of 42.2% of the variance with the three subscales explaining 28.1, 7.2 and 6.9%, respectively. All items loaded above 0.40 on their respective subscales, except item 12 (“Tingling in the hands/feet, 0.378) and item 29 (“Worry about sex life”, 0.189) (see Supplemental Table 1).

#### Rasch analysis

Overall fit of the data to the Rasch model for each subscale was given (see Supplemental Table 2). The range of item locations and item thresholds logits for all three subscales indicated that items mapped out a measurement continuum. The Symptom subscale had the widest range of item locations from −1.16 to +1.92 logits. The Healthcare support subscale had a range of item thresholds from a maximum of −3.07 to +5.28 logits. Regarding individual item fit, item 12 ‘Tingling in hands/feet’ was the only item showing misfit in the Symptoms subscale with a fit residual of +2.68. In the Emotional response subscale, three items (‘Sharing feelings with family/friends’, ‘Worry about sex life’, ‘Information about the future’) showed misfit to the Rasch model (fit indices ranged from +2.52 to +3.16). All items in the Healthcare support subscale fitted the Rasch model (see Table 3). Examination of graphical fit via item characteristic curves confirmed good fit to the Rasch model for 30/33 items, except for ‘Tingling in the hands/feet’, ‘Worry about sex life’ and ‘Information about future’ (see Supplemental Fig. 3). These show a slight under-discrimination, indicating difficulties to stratify participants according to different levels on the latent variable HRQOL.

**Table 3:** Myeloma Patient Outcome Scale item fit statistics ordered by location (*n* = 238)

Item	Label	Threshold ordering	Item location	Standard error	Item fit residual	*X* ^*2*^	*p* value	Threshold after collapsing response categories	Item fit residual after reordering	*p* value after reordering
*Subscale symptoms*										
1	Pain	√	−0.48	0.08	−0.01	1.5	0.674	–	–	–
2	Breathlessness	√	−0.44	0.09	0.65	4.6	0.201	–	–	–
3	Fatigue	√	−1.16	0.09	−1.28	7.2	0.067	–	–	–
4	Nausea	**×**	0.46	0.11	−0.49	3.7	0.294	0/1(A little + moderate)/2(severe + overwhelming)	−0.82	0.209
5	Vomiting	**×**	1.92	0.15	−1.07	4.6	0.202	0/1/2	−1.76	0.028
6	Poor appetite	**×**	1.52	0.09	−1.34	3.2	0.357	0/1/2	−1.41	0.159
7	Constipation	**×**	−0.37	0.08	−0.43	2.5	0.472	0/1/2	−0.61	0.421
8	Sore or dry mouth	√	−0.17	0.09	1.07	4.3	0.229	–	–	–
9	Drowsiness	√	−0.27	0.09	−1.13	3.7	0.290	–	–	–
10	Poor mobility	√	−0.59	0.08	−1.13	6.5	0.091	–	–	–
11	Diarrhoea	**×**	0.22	0.10	0.89	5.5	0.138	0/1/2	0.71	0.367
12	Tingling in hands/feet	√	−0.41	0.08	**2.68**	16.7	**0.001**	0/1/2	2.39	0.011
13	Difficulty remembering	**×**	−0.21	0.09	0.25	1.5	0.687	0/1/2	0.64	0.339
*Subscale emotional response*										
14	Anxiety	√	0.06	0.08	−2.18	15.3	**0.002**	0/1/2	−1.80	0.006
15	Family anxiety	√	−0.26	0.07	0.87	0.7	0.864	0/1/2	0.53	0.974
16	Depression	**×**	0.29	0.08	−0.83	7.9	0.047	0/1/2	−1.32	0.035
17	At peace	**×**	−0.69	0.08	−1.69	13.9	**0.003**	0/1/2	−1.20	0.036
18	Sharing feelings	**×**	−0.03	0.07	**2.52**	3.6	0.308	0/1/2	2.49	0.041
19	Information	**×**	0.23	0.07	−0.13	2.6	0.453	0/1/2	−1.03	0.519
20	Practical matters	**×**	0.31	0.08	0.45	1.3	0.741	0/1/2	0.88	0.624
21	Usual activities	**×**	−0.26	0.07	0.21	1.7	0.639	0/1/2	−0.21	0.705
22	Hobbies	**×**	−0.66	0.06	0.81	8.5	**0.037**	0/1/2	−0.55	0.423
23	Quality time with family/friends	√	0.26	0.08	−0.91	5.7	0.126	0/1/2	−1.06	0.301
24	Worry about sex life	**×**	0.17	0.08	**3.16**	27.6	**0.001**	0/1/2	**2.79**	**0.001**
25	Worry about infections	**×**	0.15	0.08	1.45	4.3	0.228	0/1/2	1.22	0.223
26	Worry about physical appearance	√	0.29	0.08	−0.17	0.3	0.953	0/1/2	0.13	0.402
27	Worry about financial situation	**×**	0.17	0.07	−0.02	3.0	0.391	0/1/2	0.44	0.285
28	Worry about illness worsening	√	−0.50	0.07	−1.64	8.4	**0.038**	0/1/2	−1.72	0.010
29	Coping with illness and treatment	**×**	0.41	0.09	−1.93	19.3	**0.001**	0/1/2	−2.40	0.018
33	Information about future	**×**	0.06	0.07	**2.99**	19.4	**0.001**	0/1/2	**2.79**	0.044
*Subscale: healthcare support*										
30	Contact for advice	**×**	−0.69	0.15	0.46	1.8	0.411	0/1/2/3 + 4	1.27	0.109
31	Knowledge/skill of doctors	**×**	−0.14	0.17	0.25	5.3	0.069	0/1/2/3 + 4	0.56	0.023
32	Care and respect	**×**	0.83	0.24	−0.20	4.8	0.092	0/1/2/3 + 4	0.05	0.154

Bolded values indicate fit residuals outside the recommended range of −2.5 to +2.5 or significant *X* ^*2*^-values

Regarding item response options, thresholds were ordered for 12/33 items, but for 21/33 items the 5-point scale did not work in a linear way (see Supplemental Table 2). For ten of these items, people appeared to have difficulty discriminating between the last two to three categories, thus distinguishing a moderate problem from a severe or overwhelming one. For 11 items, people seemed to have difficulty discriminating between the first two categories (‘not at all’ and ‘slight’/’moderate’). Fit for all items improved after removing extreme persons and rescaling the MyPOS items showing misfit and disordered thresholds to a 3-point Likert scale by combining categories “A little” and “Moderate”, and combining “Severe” and “Overwhelming”, the two highest response categories. After rescoring, all items on the Symptom subscale showed ordered thresholds. In the emotional subscale, item 19 (Having enough information about the illness”) and item 33 (“Having enough information about what might happen in the future”) retained disordered thresholds, as did item 32 (“Doctors/nurses show care & respect”) on the Support subscale. Chi-square test statistics and the person separation index did not improve on this last subscale after rescoring and the Support subscale does not fit the Rasch model.

Some item redundancy was present for seven pairs of items that had residual correlations exceeding *r* < 0.30 (3% of total correlations). The following item pairs showed potential redundancy: Nausea–Vomiting (*r* = 0.37), Problems with feeling at peace-Depression (*r* = 0.36), Problems with sharing feelings with family–family anxiety (*r* = 0.39), Hobbies-Usual activities (*r* = 0.36), Worry about illness worsening-Anxiety (*r* = 0.35). Two pairs of items in the Healthcare support subscale correlated highly: Contacting doctors for advice—knowledge of staff (*r* = 0.82) and Contacting doctors for advice-Doctors showing respect (*r* = 0.55).

### Floor and ceiling effects

For most items, all response options were endorsed. However, 10/33 items (‘Nausea’, ‘Vomiting’, ‘Poor appetite’, ‘Sore or dry mouth’, ‘Diarrhoea’, ‘Drowsiness’, ‘Tingling in the hands/feet’, and three items in the Healthcare support subscale) had floor effects with participants not using the two highest levels. These were also the items with the most skew. Up to 18/33 items had percentages of >50% of participants choosing the option ‘Not at all’. The MyPOS total score and subscale scores showed a normal distribution except for the Healthcare support subscale which demonstrated skew >2.5 at each time point.

**Fig. 1:**
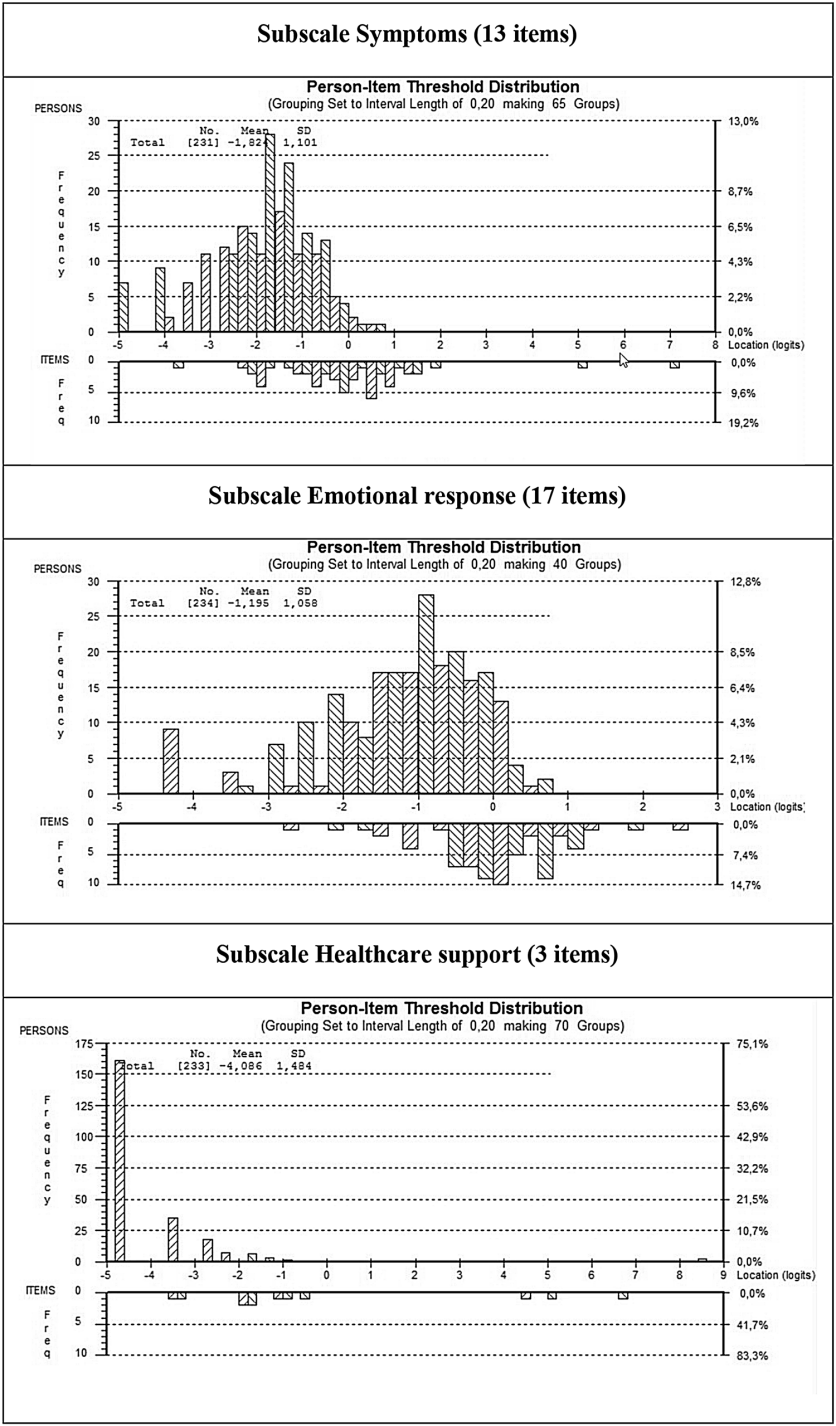
Targeting of the sample (person-item location distribution maps) for the three subscales Symptoms (*first panel*), Emotional response (*second panel*), and Healthcare Support (*third panel*) *Note* the figure shows the distribution of person measurements (*upper histogram*) against the distribution of item locations (*lower histogram*). People are located along a continuum of low quality of life (*left-hand side*) to better quality of life (*right-hand side*). Items are located relative to their difficulty: easier items (representing lesser impact on quality of life) on the *right-hand side*, and the most difficult items (required for a better quality of life) on the *left-hand side*. People outside the scales measurement range (−2 to +2 logits) indicate suboptimal scale-to-scale targeting. A ceiling effect is seen when the person locations on the *left-hand side* do not cover the item locations below, meaning items not discriminating in the portion of the sample with high quality of life

In Rasch analysis, 14 person fit residuals exceeded the recommended range of −2.5 to +2.5 (−3.68 to 3.55); implying that approximately 6% of people gave responses not in keeping with expected scores. Scale-to-scale targeting was suboptimal. Figure 1 shows the person estimation-item location distribution for the three MyPOS subscales. The sample covers the bulk of possible item locations on the MyPOS Symptom. Some mistargeting exists for the Emotional response subscale. The scale did not cover the sample in the Healthcare support scale, indicating floor effects.

### Reliability of the Myeloma Patient Outcome Scale

The Person separation indices implied good sample separation and high reliability (Supplemental Table 2), except for the Healthcare support subscale consisting of only three items. This was confirmed by values of Cronbach’s alpha that did not exceed a lower bound of 0.795.

Variance decomposition shows that the largest component is error variance. Next, variance is due to participants experiencing change between assessments (Table 4), reflected by high between-person variation and interaction terms for person x time and indicating that participants experienced different HRQOL trajectories over the period of eight months. The generalizability coefficients (Table 4) show that (a) reliability of screening was reasonable to good (*R*_IF_ 0.55 to 0.73), (b) discrimination was lower (*R*_IK_ < 0.50), except for the Healthcare support scale, (c) test–retest reliability of the MyPOS was excellent (*R*_KF_ > 0.90), (d) MyPOS can reliably measure change in individual patients over time (*R*_C_ > 0.60), except in the Healthcare support subscale (*R*_C_ = 0.42).

**Table 4:** Variance partitioning of MyPOS total and subscale scores and Generalizability reliability coefficients

Source of variance	Total MyPOS		Symptoms		Emotions and functioning		Healthcare support	
	var	%	var	%	var	%	var	%
Person	0.11	12.5	0.097	12.7	0.177	17.1	0.05	20.0
Time point	0.143	16.2	0.164	21.4	0.108	10.4	0.005	2.0
Item	0.004	0.5	0.003	0.4	0.006	0.6	0.001	0.4
Person × time point	0.2	22.7	0.178	23.3	0.202	19.5	0.021	8.4
Person × item	0.083	9.4	0.066	8.6	0.143	13.8	0.087	34.8
Time point × item	0.007	0.8	0.006	0.8	0.009	0.9	0	0.0
.Error	0.334	37.9	0.251	32.8	0.393	37.9	0.086	34.4
Total	0.881	100.0	0.765	100.0	1.038	100.0	0.25	100.0
Standard error of measurement	6.9		3.2		4.9		1.1	

Scale	RIF		RIR		RKF		RC	
	Screening		Discrimination		Test–retest reliability*		Reliability of change	
Total MyPOS	0.553		0.233		0.970		0.642	
Symptoms subscale	0.587		0.218		0.974		0.680	
Emotions subscale	0.632		0.338		0.978		0.607	
Healthcare support	0.734		0.591		0.986		0.423	

* Test–retest reliability is based on patients who indicated their QOL as stable on the global rating of change

Item invariance via DIF analysis identified the items ‘Constipation’, ‘Drowsiness’, ‘Diarrhoea’ in the Symptom subscale as unstable over time. In the Emotional response subscale, only the item ‘Worry about infections’ showed DIF. None of the items in the Healthcare support subscale showed DIF (see Supplemental Table 3).

### Responsiveness of the Myeloma Patient Outcome Scale

**Fig. 2:**
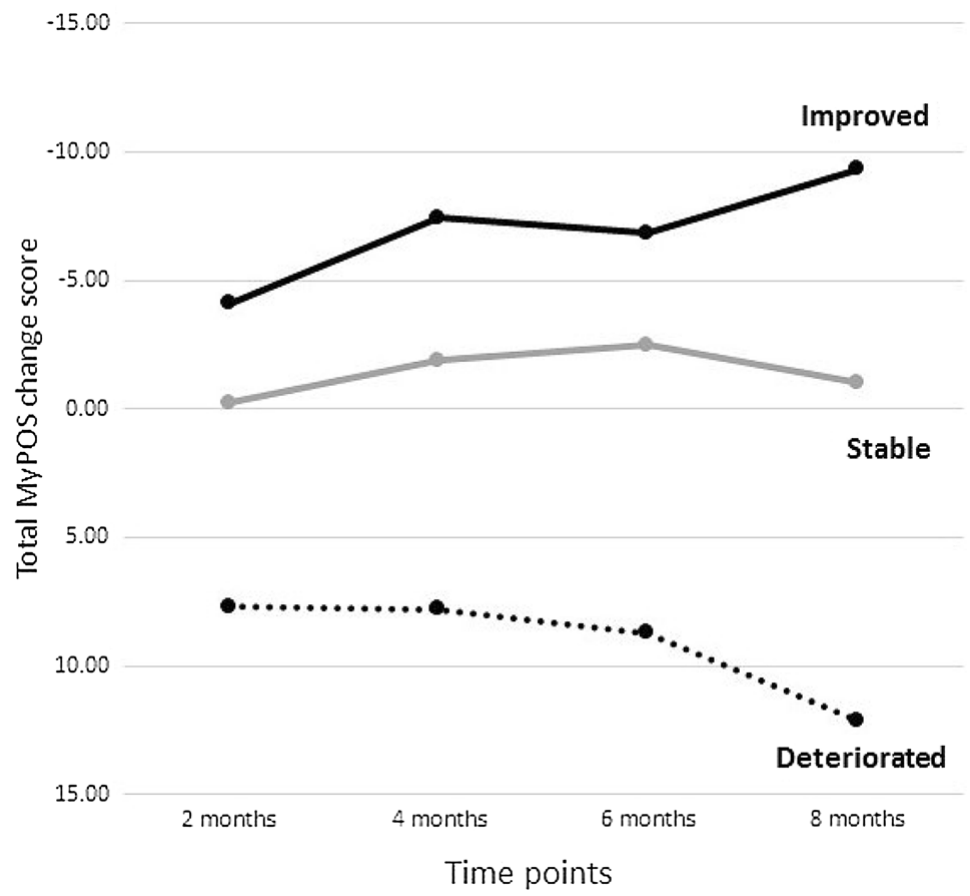
Responsiveness of the total MyPOS change score over 8 months post baseline. *Note* a negative change score on the total MyPOS denotes an improvement in quality of life

The total MyPOS score correlated moderately with the global rating scale (GRC, anchor) at every time point (range: *r* = 0.312 to *r* = 0.482). 125 participants contributed data for all five time points. Equal numbers of participants experienced a change in quality of life for the better or the worse, but the majority (about 60%) experienced no change (see Supplemental Table 4). Figure 2 shows the plotted change scores across time points. Except for the *Healthcare support* subscale, all mean change scores and corresponding confidence intervals indicated an improvement in MyPOS scores when patients classified themselves as overly improved, and a worsening of MyPOS scores when participants described their general quality of life as deteriorated.

Table 5 lists the optimal cut-off points (MIDs). For patients who reported they had improved, the MID for the total MyPOS score was 2.5. The subscale MIDs were 1.5 for Symptoms, 4.5 for Emotional response and 0.5 for Healthcare support. MIDs for deterioration were similar to those for improvement, with an MID of 4.5 for the total score and MIDs of 2.5, 3.5 and 0.5 for the subscale scores. The range of MIDs is much larger derived from the distribution-based approach, with estimates ranging from a minimum of 3.4–13.4 in the total score and 0.3–9 in the subscale scores (Table 5). Further examination of mismatch between the two methods and uncertainty around the MID revealed greater misclassification for improvement than for deterioration (see distribution graph for total MyPOS, Supplemental Fig. 3). The area under the ROC curve predicting improvement or deterioration was significantly greater than 0.5 (*p* < 0.01) for the total MyPOS change score and all subscale scores except the Healthcare support subscale.

**Table 5:** Minimal important differences calculated by using mean score changes by global rating scale, receiver-operating characteristic curve estimates and the standard deviation of baseline scores

	N	Mean changes by GRC		ROC analysis						Effect sizes			
		M_c_	95% CI	Cut-off point	Sens/Spec (%)	AUC (95% CI)	*p* value^g^	Sum of % misclassified	95% limit	SD^e,f^	Small^a^	Moderate^b^	Large^c^
Total MyPOS^d^										16.8			
Improved	22/50	8.7	(3.0, 14.3)	2.5	77/66	0.717 (0.576, 0.858)	**0.004**	56.7%	30.9		3.4	8.4	13.4
Deteriorated	21/50	−10.3	(−17.7, −2.9)	−4.5	82/57	0.719 (0.568, 0.870)	**0.004**	60.8%	18.3		−3.4	−8.4	−13.4
MyPOS symptom^d^										6.1			
Improved	23/64	3.3	(0.8, 5.9)	1.5	65/75	0.691 (0.559, 0.823)	**0.007**	59.8%	13.5		1.2	3.1	4.9
Deteriorated	26/64	−2.7	(−4.7, −0.7)	−2.5	79/57	0.687 (0.550, 0.824)	**0.006**	62.6%	6.1		−1.2	−3.1	−4.9
MyPOS emotions^d^										11.3			
Improved	25/59	6.1	(2.8, 9.5)	4.5	56/76	0.701 (0.572, 0.830)	**0.004**	67.7%	20.2		2.3	6.2	9.0
Deteriorated	24/59	−8.0	(−13.7, −2.3)	−3.5	88/54	0.691 (0.544, 0.839)	**0.006**	57.7%	15.3		−2.3	−6.2	−9.0
MyPOS support^d^										1.5			
Improved	26/78	−0.2	(−1.1, 0.8)	0.5	26/80	0.565 (0.442, 0.688)	0.322	92.3%	3.7		0.3	0.8	1.2
Deteriorated	29/78	−0.3	(−0.8, 0.2)	−0.5	78/27	0.544 (0.421, 0.667)	0.481	94.2%	1.8		−0.3	−0.8	−1.2

*Sens* sensitivity—proportions of patients correctly identified by the test as changed, *Spec* Specificity—proportions of patients correctly identified by the test as unchanged, *GRS* Global rating scale of change, *ROC* Receiver operating characteristic, *SD* standard deviation, *CI* confidence interval, *AUC* Area under the curve, Sum of % misclassified: [(1 − Sens) + (1-Spec)]

^a^Small effect size = 0.2 x SD_baseline_

^b^Medium effect size = 0.5 x SD_baseline_

^c^Large effect size = 0.8 x SD_baseline_

^d^Positive scores mean more symptoms/problems

^e^Total sample (improved, no change or deteriorated)

^f^Standard deviation of baseline scores

^g^Bold values indicate statistically significant area under the curve values

### Acceptability of frequent self-monitoring for patients

46% of participants thought the MyPOS to be a feasible tool for monitoring symptoms and problems/concerns over time. 23.9% of patients did not believe it was acceptable to complete the MyPOS regularly before clinic visits. 30% of responses were missing due to drop-out at this time. Concerns about acceptability fell into two categories: (a) those who thought it unfeasible to monitor changes because their condition changed on a daily basis and a questionnaire could not capture these minute alterations; and (b) those who felt that their clinical team monitored their condition regularly and a questionnaire would duplicate information. Linked to both of these were concerns regarding overall burden, especially when receiving treatment within a clinical trial with regular data collection, and associated cost. Positive statements included the belief that monitoring would help to focus on the symptoms and problems over time, something which these patients felt was often disregarded or overlooked in consultations: “It would help the patient to focus on their treatment, difficulties and problems. We are not always aware that some problems and side effects are related to medication and treatment and try to ignore them”. (Female participant with relapsed disease)

## Discussion

In the CTT and Rasch psychometric analysis, the MyPOS, a disease-specific measure of quality of life and palliative care concerns in multiple myeloma, presented as having adequate construct validity and reliability for certain subscales and items. For example, in the Rasch analysis items mapped out a measurement continuum in all three subscales. In terms of suitability for longitudinal monitoring, it had excellent test–retest reliability as well as reliably measuring change and being responsive. The MyPOS was able to discriminate between subgroups of patients longitudinally. However, some symptom and health care support items with floor effects, suboptimal scale-to-scale targeting and disordered thresholds point towards areas for revision. These revisions in particular concern the third subscale, *Healthcare support*, which overall had very substantial floor effects in the items, high inter-item correlations and thus item redundancy. Further targets are items in the *Emotional Response* subscale, particularly items 15 (“Family anxiety”) and 18 (“Sharing feelings with family/friends”), item 14 (“Anxiety”) and item 28 (“Worry about illness worsening”), item 21 and 22 (“Usual activities”/”Hobbies”) and items 19 (“Information about illness/treatment”) and 33 (“Information what might happen in the future”). It is worth exploring whether the MyPOS could be shortened by removing redundant items, which might also improve model fit in the factor analysis, and whether a two-factor structure (after removal of the *Healthcare Support* items) provides a better fit to the data.

Any revisions of the MyPOS must weigh information on psychometric quality with considerations of clinical utility of the item in the clinical context [60]. Revisions need to balance considerations regarding content validity, clinical usefulness and applicability of the item and take item quality into account. A systematic review [13] identified 13 HRQOL instruments validated in myeloma, most of them generic in nature (EORTC QLQ-C30, EQ-5D and 15D, FACT-G, SF-36/12). This poses a problem as generic questionnaires do not include disease-specific concerns and symptoms and are therefore less suited for validly reflecting patient experience [18]. The MyPOS was subsequently developed following extensive patient interviews to close the gaps in item coverage identified in other HRQOL instruments, and to operationalise disease-specific HRQOL according to a conceptual model developed from these qualitative interviews [35].

We argue further that for clinical applicability, considerations of test–retest reliability and responsiveness to change for enabling the valid monitoring of patients in clinical practice are paramount. However, this information is often not available for disease-specific tools in multiple myeloma. For example, an MID was only determined for the EORTC QLQ-C30 and the two health state measures EQ-5D and 15D [61, 62]. Subsequently, two new disease-specific tools, the MDASI-MM [63] and the FACT-MM [64], have been developed, but their validation has not yet been completed or has not included longitudinal validity testing to date. Another aspect lacking from validation studies is the investigation of scaling quality. One notable exception is a study exploring Mokken scaling stability of the EORTC QLQ-C30 across different subpopulations of myeloma [65]. However, this analysis did not provide in-depth information on each item and did not look at item stability in a longitudinal context. For the MyPOS, we provide both information on scaling quality and longitudinal validity.

Regarding possible revisions of the MyPOS, the measurement aim needs to be considered. For example, floor effects in gastrointestinal symptoms may be observed for most of the sample of a relatively stable myeloma population not currently undergoing anti-cancer treatment or receiving maintenance treatment only [66]. However, they are important symptoms to monitor for the clinician to make adjustments to the treatment plan should they suddenly become severe [67–70]. Inspection of the person-item threshold maps shows that it is not the items in the measure that do not cover the whole spectrum but rather the sample that did not target all the item difficulty locations. Similarly, floor effects are commonly seen in HRQOL and health satisfaction measures that are constructed with the intention of being applicable to a wide range of disease severity levels [71–73]. This is even true for disease-specific scales and was observed in the field-testing of the EORTC QLQ-MY24 [74], subsequently revised to 20 items. Floor effects in healthcare support items may reflect the finding that respondents have more positive experiences with the healthcare they received affecting their willingness to participate in studies from the outset [75]. However, while revision of the scale helped improve the fit of items in the *Symptoms* and *Emotional Response* subscale, these response scale adaptations should be performed after further qualitative, cognitive interview work [59, 76]. Another option is to extend the range of item difficulties to cover all levels of severity and impact of myeloma on HRQOL by constructing item banks and computer adaptive testing [77]. In our analysis, we tried to combine the perspectives of traditional psychometric approaches (confirmatory factor analysis, responsiveness and MID) with modern item response theory for evaluating the stringent criteria proposed by McHorney et al. [23] for longitudinal individual patient monitoring. Using the new approaches addresses shortcomings of CTT such as validating only total scores instead of single items in a measure and yielding sample-dependent results [30]. The benefits of Rasch analysis include item-level statistics and information on how items can be improved to fit the application in a specific sample [78]. Furthermore, generalizability theory [26–28] allows an exploration of sources of variation in item scores, which leads to establishing various reliability indices to distinguish different scenarios of use, i.e. using HRQOL measures for screening (single application) or for monitoring (application to track outcomes over time in an individual). This extends the limited exploration of test–retest reliability in CTT approaches [22]. The new psychometrics are proposed as extensions to the original operationalisations of measurement quality criteria that were proposed by McHorney et al. [23] in their seminal paper. They can potentially offer additional information on sources of floor & ceiling effects and, due to Rasch analysis yielding information on the full range of the construct being measured, sources of problems with the coverage of constructs and diverse patient groups. The same is true for Generalizability analysis that provides a fine-grained picture of sources of measurement error beyond the random measurement error and can therefore help understand problems with precision of measurement in the cross-sectional and the longitudinal application [27, 28]. However, especially the latter approach to reliability assessment and the indices proposed by Cranford et al. [29] are limited by not being used widely in the literature which makes their interpretation difficult. For example, it is not clear whether thresholds for acceptable ICC estimates as proposed by McHorney et al. [23] are applicable for the screening, discrimination and reliable change index proposed in this paper [29]. Further research is needed to explore this issue. Moreover, we used Cranford et al.’s [29] method in a situation of a less intensive longitudinal design, with far less frequent measurement than was employed in their diary study. Therefore, the analysis of sources of variation stemming from different time points is not as detailed as in their original analysis.

Applying the framework of quality criteria for individual patient-monitoring to the MyPOS yields the following assessment of its suitability for this application. Regarding (i) practical features, survey administration is well below 15 min [15], however, the number of items is rather high for a clinically applicable tool [18]. The analysis of breadth of health measured (ii) yields good dimensionality of the measure and coverage of all aspects of disease-related QOL according to the theoretical model [15], however, scale revisions indicated by low factor loadings, item redundancy and poor fit of the *Healthcare Support* subscale call for further exploration of dimensionality. Criterion (iii), the depth of health measured, was partially fulfilled with floor effects showing in 10/33 items and person-item targeting analysis within Rasch modelling suggesting further analysis in more severely affected samples. Criteria (iv) and (v) pertaining to reliability were assessed slightly differently by extending suggested analyses of Cronbach’s alpha for cross-sectional reliability and test–retest reliability by Rasch analysis and Generalizability theory, and by omitting standard error of measurement as a quality criterion. Although the actual size of the coefficient that should be obtained is unclear, the rigorous criterion for reliability (>0.95) set by McHorney et al. [23] was achieved for all subscales in longitudinal analysis, but not for cross-sectional reliability (screening & discrimination application, Cronbach’s alpha). Validity (vi) in terms of cross-sectional construct validity and responsiveness to change yielded good sensitivity to change values. Further convergent and divergent validity assessment is reported in the initial validation of the MyPOS [15].

One of the most important features that makes a scale suitable for monitoring purposes is its responsiveness to change [19]. Our MIDs for improvement and deterioration were smaller than the MIDs reported by Kvam and colleagues for the EORTC QLQ-C30 for patients with multiple myeloma [62]. Their MIDs range from 6 to 17 points for improvement and 12–27 points for deterioration, a small to medium change [62]. This discrepancy might arise from the different nature of the QLQ-C30, a generic measure, with absolute higher values of meaningful change [78–81]. The large baseline standard deviations and the amount of misclassification that was seen imply that not enough patients in our sample experienced a substantial change and that there further exists imprecision in the anchor in classifying participants into improved and deteriorated. This is a commonly reported problem with the ROC-curve based method of MID [54, 82] which, as a diagnostic approach, would require a bias-free and precise gold-standard anchor. However, in the absence of guidance regarding construction of global rating scales this situation might not easily be rectified.

The first limitation of our study is the use of consecutive enrolment, resulting in a convenience sample. The strength lies in its greater clinical representativeness that counteracts the effect of sampling younger and fitter patients if validation is part of a clinical trial [66, 83]. However, since we recruited from outpatient clinics or day centres, we potentially missed patients feeling too unwell to participate in a longitudinal survey. This was the first study to use Generalizability theory. This approach for evaluating sensitivity to change normally requires frequent assessments [29]. However, due to patient burden this was not feasible. The reliability coefficients may be an underestimation of the true longitudinal reliability of the MyPOS. Furthermore, since this approach is relatively new, there are no guidelines as to the size of the coefficients. Confirmatory factor analysis used the DWLS approach to account for non-normality and the ordinal nature of the response scale in the MyPOS. However, although this approach has been reported as robust in samples of above 200, a caveat is its use in situations were missing data is missing not at random [84]. Baseline data was used for confirmatory factor analysis with missingness likely not due to systematic item nonresponse or non-random mechanisms. However, low factor loadings of some items might be due to systematic bias, i.e. for item 24 “Worry about sex life”, with effect on model fit. Different groupings of functioning items on subscales in the reported factor analysis compared to the initial factor analysis reported in Osborne et al. [15] are most likely due to changing descriptive labels of the rating scale of the symptom items to adapt the MyPOS to the overall item and scaling format of the IPOS [32, 36], of which it is a module. In the adapted version of the MyPOS, the rating scale for the symptoms only lists the severity of impairment but not the added descriptor “impaired activity or concentration”. This change might have affected other aspects of construct validity, which likely necessitates a re-validation of aspects of construct validity of the symptom subscale. For the anchor-based MID approach, there is no consensus for the amount of categories and the exact phrasing of the global rating scale of change. Authors have used 3-point [56] to 15-point scales [85]. We tried to balance the potential lack of sensitivity of fewer response options with the need to arrive at a valid measurement of change presenting only so many levels which patients can adequately discriminate. Since we asked patients to compare a change in their condition always to the first assessment, recall bias may have affected at least part of the sample. Furthermore, the wording of the rating scale might not present a valid global assessment of change in quality of life as operationalised in the multi-dimensional, disease-specific MyPOS. The validity of the global rating of change as a criterion for anchor-based derivation of the MID is further pulled into question by the relatively low correlation between anchor and change scores and the MID not exceeding the SEM in all subscales.

## Conclusion

This analysis supported the responsiveness and test–retest reliability of the MyPOS, using a multi-centre outpatient sample of patients at different disease stages. Additional derivation of the MID for use in individual patient care and exploration of valid anchors of global change are needed. Modifications to the scoring format and potential removal of the Healthcare Support subscale may be warranted, subject to further testing. The study was the first to apply Generalizability theory to establish test–retest reliability and stability of scores in frequent measurements in medicine.

## Electronic supplementary material

Below is the link to the electronic supplementary material.

Supplementary material 1 (DOCX 1119 kb)
