# Supplementary material for: Longitudinal validity and reliability of the Myeloma Patient Outcome Scale (MyPOS) was established using traditional, generalizability and Rasch psychometric methods
Source: Qual Life Res. 2017 Jul 27;26(11):2931–47. doi: 10.1007/s11136-017-1660-z (PMC5655545; doi:10.1007/s11136-017-1660-z)
Supplement: Supplementary file 1 — Supplementary material 1 (DOCX 1119 kb) [file 11136_2017_1660_MOESM1_ESM.docx]

**SUPPLEMENTAL MATERIALS**

**Longitudinal validity and reliability of the Myeloma Patient Outcome scale was established using traditional, generalizability and Rasch psychometric methods**

**C**hristina Ramsenthaler, Wei Gao, Richard J. Siegert, Stephen A. Schey, Polly M. Edmonds, Irene J. Higginson

**Supplemental Table 1** Factor loadings of the MyPOS and fit statistics for confirmatory factor analysis

| **Item no.** | **Description** | **Symptoms** | **Emotions** | **Support** |
| --- | --- | --- | --- | --- |
| 1 | Pain | .702 |  |  |
| 2 | Shortness of breath | .560 |  |  |
| 3 | Fatigue | .753 |  |  |
| 4 | Nausea | .783 |  |  |
| 5 | Vomiting | .730 |  |  |
| 6 | Appetite loss | .710 |  |  |
| 7 | Constipation | .573 |  |  |
| 8 | Mouth problems | .527 |  |  |
| 9 | Drowsiness | .670 |  |  |
| 10 | Poor mobility | .779 |  |  |
| 11 | Diarrhoea | .458 |  |  |
| 12 | Tingling | .378 |  |  |
| 13 | Remembering | .618 |  |  |
| 14 | Anxiety |  | .813 |  |
| 15 | Family anxiety |  | .661 |  |
| 16 | Depression |  | .802 |  |
| 17 | At peace |  | .831 |  |
| 18 | Sharing feelings |  | .558 |  |
| 19 | Information |  | .616 |  |
| 20 | Practical matters |  | .533 |  |
| 21 | Usual activities |  | .798 |  |
| 22 | Hobbies |  | .791 |  |
| 23 | Quality time |  | .753 |  |
| 24 | Sex |  | .189 |  |
| 25 | Infections |  | .446 |  |
| 26 | Appearance |  | .608 |  |
| 27 | Finances |  | .505 |  |
| 28 | Illness |  | .763 |  |
| 29 | Coping |  | .844 |  |
| 30 | Advice |  |  | .766 |
| 31 | Knowledge |  |  | .969 |
| 32 | Care and respect |  |  | .883 |
| 33 | Future |  | .486 |  |
|  | Variance explained | 28.1% | 7.2% | 6.9% |
| **Confirmatory factor analysis** | | | | |
| *χ^2^ (DWLS estimator)* | | 873.182 | | |
| *χ^2^* p-value | | <0.001 | | |
| *χ^2^*/ df | | 1.774 | | |
| CFI (>0.90) | | 0.942 | | |
| TLI ( | | 0.937 | | |
| NFI (>0.95) | | 0.873 | | |
| NNFI (>0.95) | | 0.937 | | |
| RMSEA (90% CI) (<0.06) | | 0.056 (0.050-0.063) | | |

**Abbreviations**: CFI, comparative fit index; df, degrees of freedom; NFI, normal fit index; NNFI, non-normal fit index; RMSEA, root mean square error of approximation; MyPOS, Myeloma Patient Outcome Scale.

**Note**: Thresholds for fit indices are indicated in brackets.

**Supplemental Table 2** Rasch model fit for each subscale with item and person location fit statistics

| Measurement characteristic | **Symptom subscale (13 items)** | **Emotional response subscale (17 items)** | **Healthcare support subscale (3 items)** |
| --- | --- | --- | --- |
| **Item locations** |  |  |  |
| Mean (SD) | 0 (0.858) | 0 (0.348) | 0 (0.774) |
| Range | -1.16 to 1.92 | -0.69 to 0.41 | -0.69 to 0.83 |
| **Thresholds** |  |  |  |
| Range | -2.49 to 5.62 | -2.19 to 1.86 | -3.07 to 5.28 |
| Fit residuals: Mean (SD) | -0.102 (1.191) | 0.175 (1.66) | 0.170 (0.338) |
| Skewness | 0.812 | 0.353 | -0.221 |
| **Person measures** |  |  |  |
| Mean (SD) | -1.824 (1.101) | -1.195 (1.058) | -4.086 (1.484) |
| Range | -4.89 to 0.63 | -4.33 to 0.69 | -4.74 to 8.42 |
| Fit residuals: Mean (SD) | -0.203 (0.875) | -0.159 (1.163) | -0.183 (0.621) |
| Skewness | 0.345 | -0.176 | -0.146 |
| Person separation index | 0.804 | 0.834 | 0.127 |
| **Overall fit** |  |  |  |
| *X^2^* | 65.651 | 143.74 | 11.887 |
| p-value | 0.005 | 0.001 | 0.065 |
| RMSEA (90% CI) | 0.054 (0.038, 0.067) | 0.088 (0.074, 0.100) | 0.064 (0.049, 0.076) |

**Note:** RMSEA was calculated according to the formula √ Max ([(X^2^/df)-1/(N-1)], 0) [82].

**Abbreviations**: CI, confidence interval; sd, standard deviation; RMSEA, root mean square error of approximation.

**Supplemental Table 3** Differential item functioning for assessing reliability

|  | **MyPOS items** | **Time (uniform DIF)** | | | **Time by class interval (non-uniform DIF)** | | |
| --- | --- | --- | --- | --- | --- | --- | --- |
|  |  | **F** | **df** | **p** | **F** | **df** | **p** |
| 1 | Pain | 0.55 | 4 | 0.698 | 1.33 | 36 | 0.093 |
| 2 | Breathlessness | 1.85 | 4 | 0.115 | 0.82 | 36 | 0.762 |
| 3 | Fatigue | 1.40 | 4 | 0.230 | 1.05 | 36 | 0.378 |
| 4 | Nausea | 1.21 | 4 | 0.304 | 1.25 | 36 | 0.145 |
| 5 | Vomiting | 0.73 | 4 | 0.570 | 1.07 | 36 | 0.356 |
| 6 | Poor appetite | 0.35 | 4 | 0.842 | 0.89 | 36 | 0.645 |
| 7 | Constipation | 0.43 | 4 | 0.784 | 1.66 | 36 | 0.009 |
| 8 | Sore or dry mouth | 1.03 | 4 | 0.388 | 1.33 | 36 | 0.090 |
| 9 | Drowsiness | 31.09 | 4 | **0.001** | 1.22 | 36 | 0.173 |
| 10 | Poor mobility | 1.85 | 4 | 0.116 | 1.22 | 36 | 0.170 |
| 11 | Diarrhoea | 33.40 | 4 | **0.001** | 1.04 | 36 | 0.406 |
| 12 | Tingling in hands/feet | 0.73 | 4 | 0.567 | 0.86 | 36 | 0.690 |
| 13 | Difficulty remembering | 1.01 | 4 | 0.399 | 0.82 | 36 | 0.763 |
| 14 | Anxiety | 0.44 | 4 | 0.773 | 0.91 | 36 | 0.605 |
| 15 | Family anxiety | 0.95 | 4 | 0.430 | 1.02 | 36 | 0.424 |
| 16 | Depression | 0.91 | 4 | 0.454 | 0.93 | 36 | 0.585 |
| 17 | At peace | 0.41 | 4 | 0.794 | 0.96 | 36 | 0.531 |
| 18 | Sharing feelings | 0.77 | 4 | 0.540 | 1.03 | 36 | 0.419 |
| 19 | Information | 1.28 | 4 | 0.273 | 0.80 | 36 | 0.786 |
| 20 | Practical matters | 0.63 | 4 | 0.634 | 1.18 | 36 | 0.216 |
| 21 | Usual activities | 1.01 | 4 | 0.401 | 0.78 | 36 | 0.815 |
| 22 | Hobbies | 0.33 | 4 | 0.855 | 1.38 | 36 | 0.067 |
| 23 | Quality time with family and friends | 0.53 | 4 | 0.709 | 1.00 | 36 | 0.464 |
| 24 | Worry about sex life | 0.67 | 4 | 0.610 | 1.32 | 36 | 0.099 |
| 25 | Worry about infections | 3.65 | 4 | 0.005 | 0.74 | 36 | 0.860 |
| 26 | Worry about physical appearance | 0.45 | 4 | 0.771 | 0.66 | 36 | 0.933 |
| 27 | Worry about financial situation | 0.61 | 4 | 0.654 | 0.89 | 36 | 0.647 |
| 28 | Worry about illness worsening | 2.12 | 4 | 0.076 | 0.93 | 36 | 0.576 |
| 29 | Coping with illness and treatment | 2.12 | 4 | 0.075 | 0.95 | 36 | 0.538 |
| 33 | Information about future | 0.99 | 4 | 0.411 | 0.76 | 36 | 0.836 |
| 30 | Contact for advice | 0.97 | 4 | 0.420 | 0.55 | 15 | 0.909 |
| 31 | Knowledge/skill of doctors | 0.37 | 4 | 0.828 | 0.54 | 16 | 0.922 |
| 32 | Care and respect | 1.14 | 4 | 0.335 | 0.62 | 16 | 0.864 |

**Note:** Bolded values indicate significant items with differential item functioning/instability over time.

**Abbreviations:** df, degrees of freedom; DIF, differential item functioning; MyPOS, Myeloma Patient Outcome Scale

**Supplemental Table 4** Changes in scores between baseline and assessments 2, 3, 4 and 5 for the total MyPOS score and its subscales. Presented are mean change scores and the standard deviation of changes.

|  | **Change TP1-TP2** | | |  | **Change TP1-TP3** | | |  | **Change TP1-TP4** | | |  | **Change TP1-TP5** | | |
| --- | --- | --- | --- | --- | --- | --- | --- | --- | --- | --- | --- | --- | --- | --- | --- |
| **MyPOS Total Score** | | | | | | | | | | | | | | | |
| **My QOL has…** | **n** | **M_c_** | **SD_c_** |  | **n** | **M_c_** | **SD_c_** |  | **n** | **M_c_** | **SD_c_** |  | **n** | **M_c_** | **SD_c_** |
| Improved | 14 | 5.7 | 10.7 |  | 22 | 8.0 | 9.3 |  | 16 | 9.6 | 13.3 |  | 22 | 8.7 | 13.5 |
| No change | 90 | 0 | 10.1 |  | 69 | 2.3 | 9.5 |  | 64 | 1.9 | 11.2 |  | 50 | 0.9 | 7.6 |
| Got worse | 11 | -8.4 | 6.9 |  | 13 | 3.8 | 13.4 |  | 21 | -8.2 | 14.5 |  | 21 | -10.3 | 17.4 |
| Missing | 84 | -7.6 | 19.7 |  | 67 | -6.2 | 9.6 |  | 49 | -3.1 | 11.5 |  | 32 | -7.5 | 11.3 |
| Rho* | 0.33 |  |  |  | 0.40 |  |  |  | 0.40 |  |  |  | 0.45 |  |  |
| **MyPOS Symptom subscale** | | | | | | | | | | | | | | | |
|  | **n** | **M_c_** | **SD_c_** |  | **n** | **M_c_** | **SD_c_** |  | **n** | **M_c_** | **SD_c_** |  | **n** | **M_c_** | **SD_c_** |
| Improved | 15 | 2.5 | 6.9 |  | 25 | 2.8 | 5.8 |  | 20 | 2.6 | 4.3 |  | 23 | 3.3 | 6.2 |
| No change | 116 | -0.2 | 4.2 |  | 90 | 1.0 | 3.9 |  | 74 | 1.0 | 5.0 |  | 64 | 0.2 | 3.6 |
| Got worse | 17 | -3.5 | 4.7 |  | 17 | -2.2 | 5.2 |  | 30 | -2.5 | 5.4 |  | 26 | -2.7 | 5.3 |
| Missing | 51 | -2.0 | 8.6 |  | 39 | -2.5 | 4.5 |  | 26 | -1.7 | 5.2 |  | 12 | -3.5 | 6.1 |
| Rho | 0.26 |  |  |  | 0.27 |  |  |  | 0.31 |  |  |  | 0.40 |  |  |
| **MyPOS Emotional response subscale** | | | | | | | | | | | | | | | |
|  | **n** | **M_c_** | **SD_c_** |  | **n** | **M_c_** | **SD_c_** |  | **n** | **M_c_** | **SD_c_** |  | **n** | **M_c_** | **SD_c_** |
| Improved | 18 | 2.4 | 8.0 |  | 26 | 4.7 | 6.4 |  | 19 | 5.4 | 9.2 |  | 25 | 6.1 | 8.6 |
| No change | 109 | 0.6 | 7.5 |  | 84 | 1.4 | 7.2 |  | 71 | 1.0 | 8.2 |  | 59 | 0.8 | 5.9 |
| Got worse | 14 | -5.0 | 5.8 |  | 15 | -2.6 | 10.6 |  | 23 | -5.7 | 10.5 |  | 24 | -8.0 | 14.2 |
| Missing | 58 | -4.2 | 10.5 |  | 46 | -2.8 | 6.3 |  | 37 | -1.6 | 8.9 |  | 17 | -3.5 | 6.1 |
| Rho | 0.20 |  |  |  | 0.28 |  |  |  | 0.35 |  |  |  | 0.41 |  |  |
| **MyPOS Healthcare support subscale** | | | | | | | | | | | | | | | |
|  | **n** | **M_c_** | **SD_c_** |  | **n** | **M_c_** | **SD_c_** |  | **n** | **M_c_** | **SD_c_** |  | **n** | **M_c_** | **SD_c_** |
| Improved | 19 | -0.1 | 0.4 |  | 27 | 0.2 | 0.9 |  | 24 | 0 | 0.7 |  | 26 | -0.2 | 2.3 |
| No change | 137 | 0.2 | 2.0 |  | 107 | 0.3 | 1.8 |  | 85 | 0.3 | 2.1 |  | 58 | 0.1 | 2.3 |
| Got worse | 20 | -0.8 | 1.4 |  | 19 | -0.3 | 1.6 |  | 31 | -0.3 | 1.1 |  | 29 | -0.3 | 1.3 |
| Missing | 23 | -0.1 | 1.4 |  | 18 | -0.2 | 0.7 |  | 10 | 0.1 | 0.5 |  | 12 | 0.1 | 0.3 |
| Rho | 0.16 |  |  |  | 0.12 |  |  |  | 0.14 |  |  |  | 0.14 |  |  |

*Spearman’s Rho shows the correlation between the change scores and the anchor at each time point. The correlation between the global change rating and the baseline values were: 0.66 (Total MyPOS), 0.23 (Symptoms subscale), 0.59 (Emotional response subscale), and 0.24 (Healthcare support subscale). **Abbreviations:** Mc, mean change score; SDc, standard deviation of change score; QOL, quality of life; MyPOS, Myeloma Patient Outcome Scale; TP, time point.

Supplemental Figure 1 The original version of the Myeloma Patient Outcome Scale (MyPOS) before it was adapted to the IPOS format. All questions are preceded by “Over the past week…”.

| **1** | What are your main problems or concerns at the moment? | [Open question with three empty boxes for respondent to complete, numbered 1-3] | | | | |
| --- | --- | --- | --- | --- | --- | --- |
| **2** | Below is a list of symptoms, which you may or may not have experienced. For each symptom please tick one box that best describes how it has affected you over the past week: | Not at all | Slightly | Moderately | Severely | Over-whelmingly |
|  |  | I have not had this symptom in the past week | Little or no effect on activities or concen-tration | Some effect on activities or concen-tration | Marked effect on activities or concen-tration | Unable to think of anything  else |
| **a** | Pain |  |  |  |  |  |
| **b** | Fatigue or lack of energy |  |  |  |  |  |
| **c** | Shortness of breath |  |  |  |  |  |
| **d** | Diarrhoea |  |  |  |  |  |
| **e** | Constipation |  |  |  |  |  |
| **f** | Nausea (feeling like you are going to be sick) |  |  |  |  |  |
| **g** | Vomiting (being sick) |  |  |  |  |  |
| **h** | Mouth problems |  |  |  |  |  |
| **i** | Poor mobility |  |  |  |  |  |
| **j** | Tingling in the hands and / or feet |  |  |  |  |  |
| **k** | Difficulty remembering things |  |  |  |  |  |
| **l** | Please list any other symptoms not mentioned above, and tick one box to show how they have affected you over the past week: | [Three boxes beneath symptoms list for respondent to add additional symptoms, numbered 1-3] | | | | |
| **3** | Have you been able to carry out your usual activities without help from others? | Yes, as much as I wanted | Most of the time | Sometimes | Occasionally | No, not at all |
| **4** | Have you been able to pursue your hobbies and leisure activities? |  |  |  |  |  |
| **5** | Have you been able to spend quality time with family and friends? |  |  |  |  |  |
| **6** | Have you been worrying about your sex life? | No, not at all | Occasionally | Sometimes | Most of the time | Yes, always |
| **7** | Have you been feeling depressed? |  |  |  |  |  |
| **8** | Have you been feeling anxious or worried about your illness or treatment? |  |  |  |  |  |
| **9** | Have you been worrying about infections? |  |  |  |  |  |
| **10** | Have you been worrying about your physical appearance? |  |  |  |  |  |
| **11** | Have you been worrying about your financial situation? |  |  |  |  |  |
| **12** | Have you been worrying that your illness will get worse? |  |  |  |  |  |
| **13** | Have you felt able to cope with your illness and treatment? | Yes, always | Most of the time | Sometimes | Occasionally | No, not at all |
| **14** | Are you able to contact your doctors or nurses for advice if needed? |  |  |  |  |  |
| **15** | Do your doctors and nurses show a good standard of knowledge skill when treating you? |  |  |  |  |  |
| **16** | Do your doctors and nurses show care and respect when treating you? |  |  |  |  |  |
| **17** | Do you have enough information about your illness and treatment? | Enough Information | Information received | Information received | Very little information | No information received |
| **18** | Do you have enough information about what might happen to you in the future? | the right amount for me | but hard to understand | but would like more | and would like more | and would like information |

Supplemental Figure 2 The modified version of the MyPOS after adaptation to the IPOS format.

| **1** | What are your main problems or concerns at the moment? | [Open question with three empty boxes for respondent to complete, numbered 1-3] | | | | |
| --- | --- | --- | --- | --- | --- | --- |
| **2** | Below is a list of symptoms, which you may or may not have experienced. For each symptom please tick one box that best describes how it has affected you over the past week: | | | | | |
| **a** | Pain | Not at all | Slightly | Moderately  Response options – impact on activity or concentration deleted | Severely | Over-whelming-ly |
| **b** | Shortness of breath |  |  |  |  |  |
| **c** | ***Weakness*** or lack of energy |  |  |  |  |  |
| **d** | Nausea (feeling like you are going to be sick) |  |  |  |  |  |
| **e** | Vomiting (being sick)  Reordering and extension by general IPOS symptoms |  |  |  |  |  |
| **f** | **Poor appetite** |  |  |  |  |  |
| **g** | Constipation |  |  |  |  |  |
| **h** | ***Sore or dry mouth*** |  |  |  |  |  |
| **i** | **Drowsiness** |  |  |  |  |  |
| **j** | Poor mobility |  |  |  |  |  |
| **k** | Diarrhoea |  |  |  |  |  |
| **l** | Tingling in the hands and / or feet |  |  |  |  |  |
| **m** | Difficulty remembering things |  |  |  |  |  |
| **n** | Please list any other symptoms not mentioned above, and tick one box | [Three boxes beneath symptoms list for respondent to add additional symptoms, numbered 1-3] | | | | |
| **3** | ***Have you been feeling anxious or worried about your illness or treatment?*** | No, not at all | Occasionally  Bolded items are new items from IPOS  Bolded items in italics are IPOS items that had already been part of the original MyPOS. These were moved to the second page. | Sometimes | Most of the time | Yes, always |
| **4** | **Over the past week, have any of your family or friends been anxious or worried about you?** |  |  |  |  |  |
| **5** | ***Have you been feeling depressed?*** |  |  |  |  |  |
| **6** | **Have you felt at peace?** | Yes, always | Most of the time | Sometimes | Occasionally | No, not at all |
| **7** | **Have you been able to share how you are feeling with your family or friends?** |  |  |  |  |  |
| **8** | ***Have you had as much information as you wanted?*** |  |  |  |  |  |
| **9** | **Have any practical matters resulting from your illness been addressed? (such as financial or personal)** | No pro-blems | Problems being addressed | Problems partly addressed | Problems hardly addressed | Problems not addressed |
| **10** | Have you been able to carry out your usual activities without help from others? | Yes, as much as I wanted | Most of the time | Sometimes | Occasio-nally | No, not at all |
| **11** | Have you been able to pursue your hobbies and leisure activities? |  |  |  |  |  |
| **12** | Have you been able to spend quality time with family and friends? |  |  |  |  |  |
| **13** | Have you been worrying about your sex life? | We would like you to answer this question whether or not you are sexually active. If you would prefer not to answer please tick here: | | | | |
|  | Have you been worrying about infections |  |  |  |  |  |
| **14** | Have you been worrying about your physical appearance? | No, not at all | Occasionally  Remaining original MyPOS items were moved to a third page. | Sometimes | Most of the time | Yes, always |
| **15** | Have you been worrying about your financial situation? |  |  |  |  |  |
| **16** | Have you been worrying that your illness will get worse? |  |  |  |  |  |
| **18** | Have you felt able to cope with your illness and treatment? | Yes, always | Most of the time | Sometimes | Occasionally | No, not at all |
| **19** | Are you able to contact your doctors or nurses for advice if needed? |  |  |  |  |  |
| **20** | Do your doctors and nurses show a good standard of knowledge skill when treating you? |  |  |  |  |  |
| **21** | Do your doctors and nurses show care and respect when treating you? |  |  |  |  |  |
| **22** | Do you have enough information about your illness and treatment? |  |  |  |  |  |

**Supplemental Figure 3** Item characteristic curves for all 33 MyPOS items

Item characteristic curves plot responses predicted by the Rasch model (curve) and observed responses for all the different levels of quality of life and palliative care concerns in multiple myeloma (the measurement continuum). The available responses are 0 ‘not at all‘, 1 ‘slight‘, 2 ’moderate‘, 3 ’severe‘ and 4 ‘overwhelming‘. The observed mean scores are plotted according to levels of quality of life with the participants with the lowest quality of life represented on the left-hand side and those with the highest observed level of quality of life represented on the right-hand side. Poor graphical fit to the Rasch model is apparent when the plotted observed means (dots) do not follow the continuous line. Items 12 ‘Tingling in the hands/feet‘, 24 ‘Worry about sex life’ and 33 ‘Information about future’ show a slight under-discrimination (also indicated by the positive fit residual for these items which is >2.5), in which participants with a higher level of quality of life report more difficulty with these areas than would be expected by the Rasch model, and participants with a lower quality of life report less difficulty with these items than would be expected.

| **1 Pain** | | **2 Breathlessness** | |
| --- | --- | --- | --- |
| **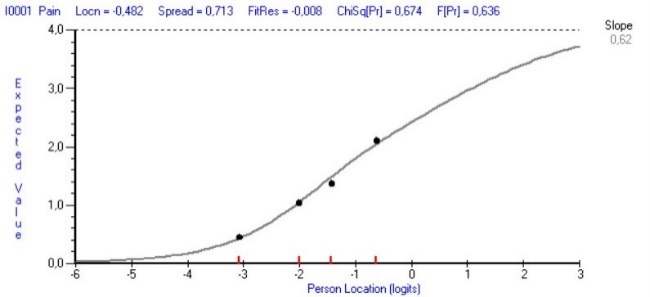** | | **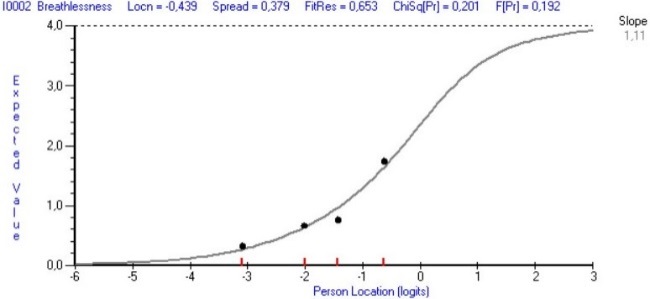** | |
| **3 Fatigue** | | **4 Nausea** | |
| **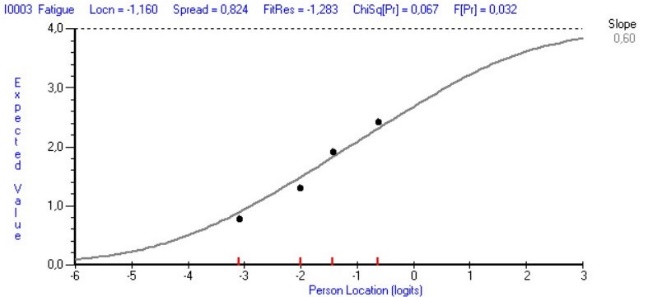** | | **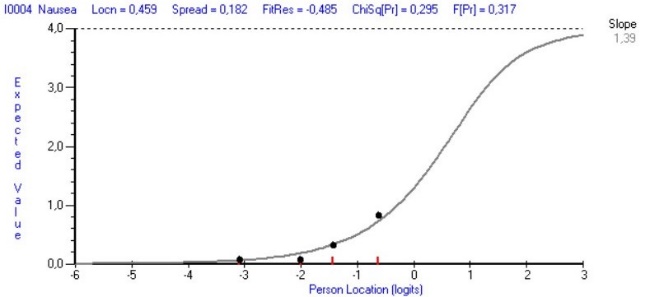** | |
| **5 Vomiting** | | **6 Poor appetite** | |
| **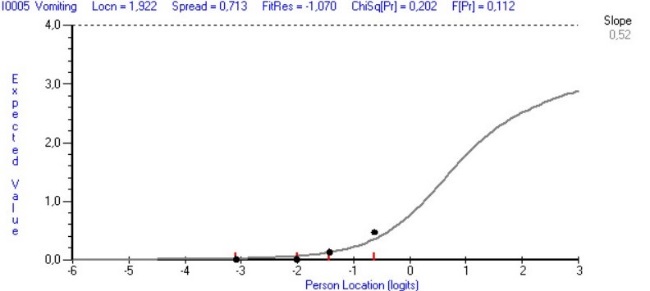** | | **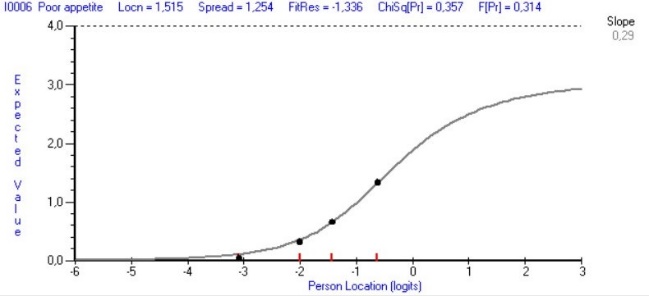** | |
| **7 Constipation** | | **8 Sore or dry mouth** | |
| **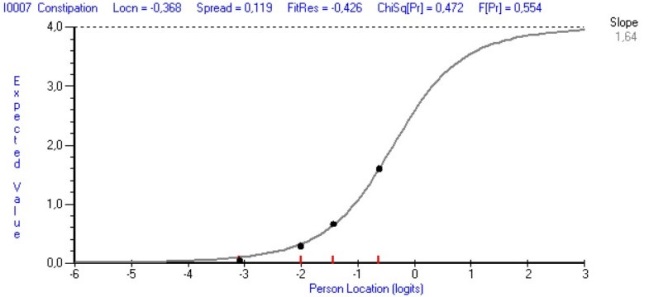** | | **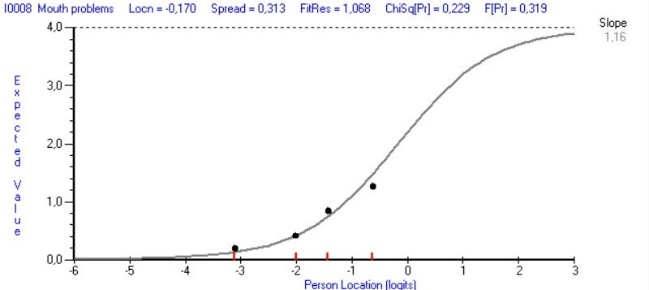** | |
| **9 Drowsiness** | | **10 Poor mobility** | |
| **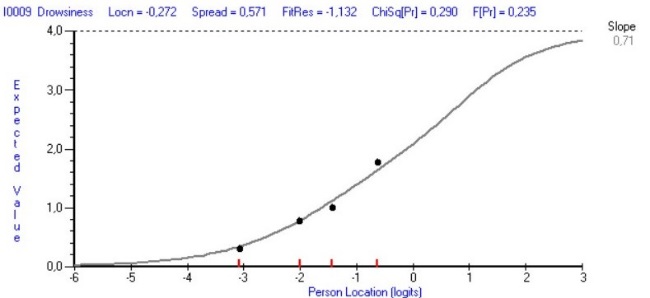** | | **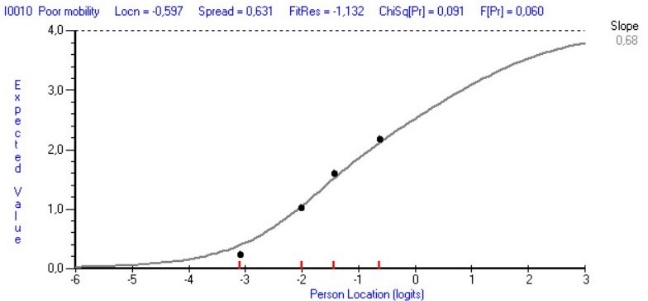** | |
| **11 Diarrhoea** | | **12 Tingling in hands/feet** | |
| **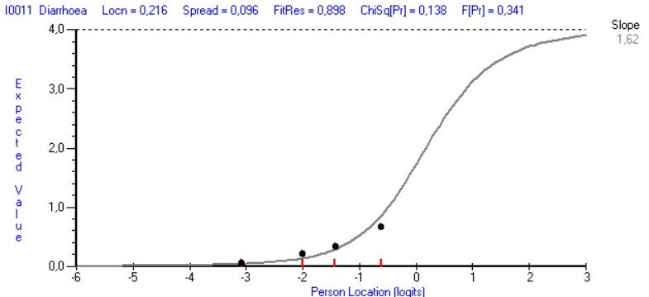** | | **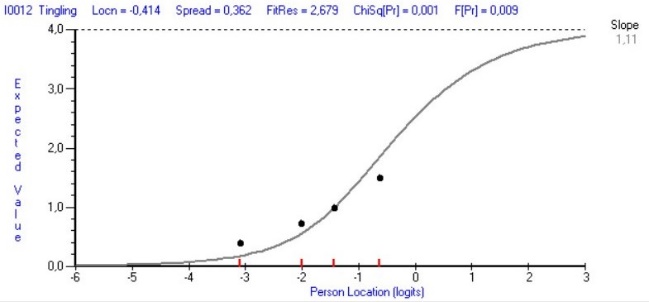** | |
| **13 Difficulties remembering** | |  | |
| **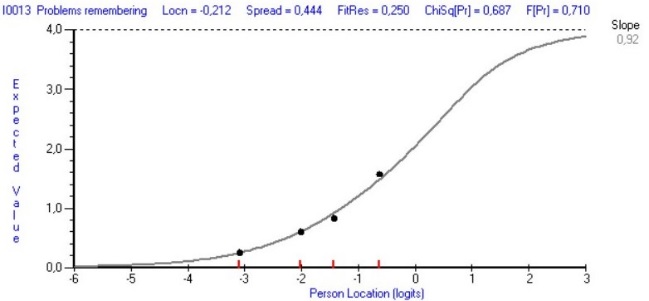** | |  | |
| **SUBSCALE EMOTIONAL FUNCTIONING** | | | |
| **14 Anxiety** | **15 Family anxiety** | |  |
| **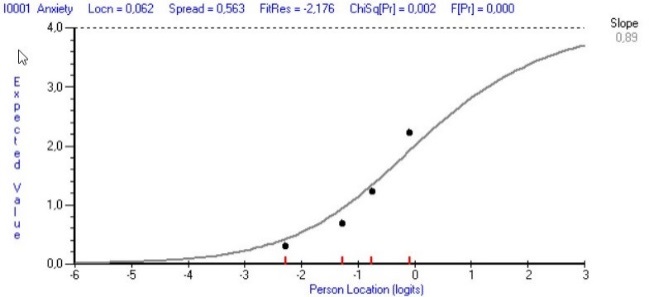** | **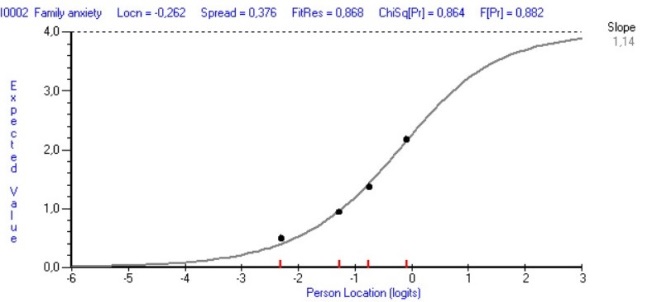** | |  |
| **16 Depression** | **17 Feeling at peace** | |  |
| **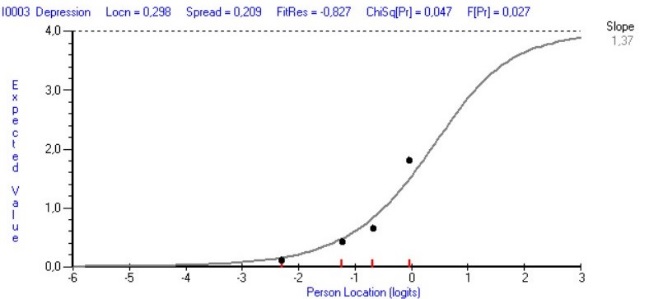** | **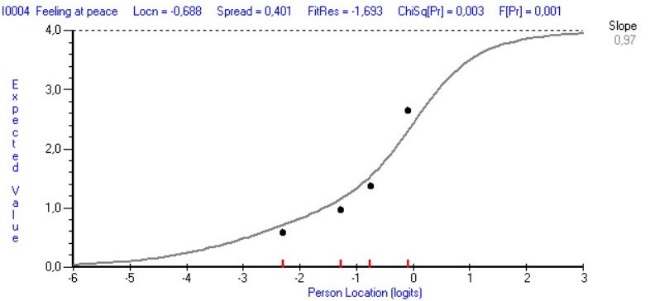** | |  |
| **18 Sharing with family/friends** | **19 Information** | |  |
| **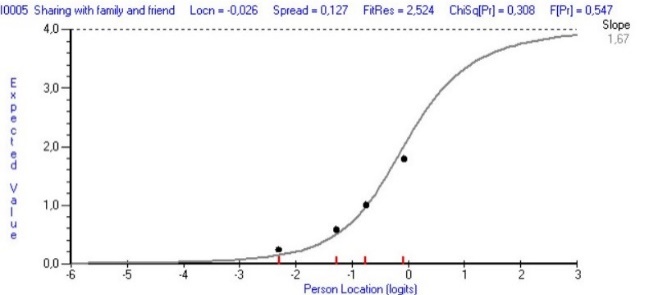** | **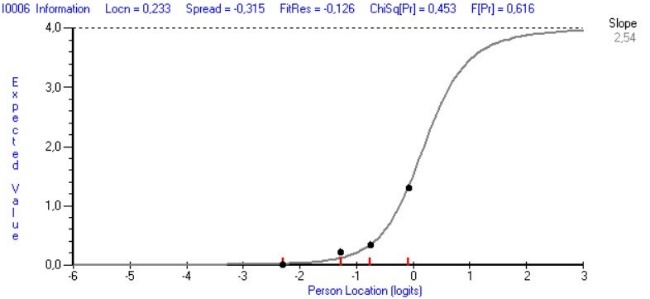** | |  |
| **20 Practical matters** | **21 Usual activities** | |  |
| **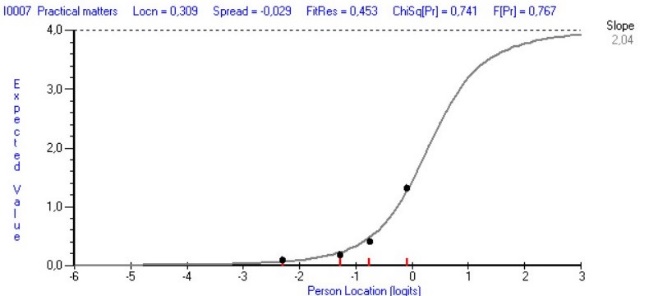** | **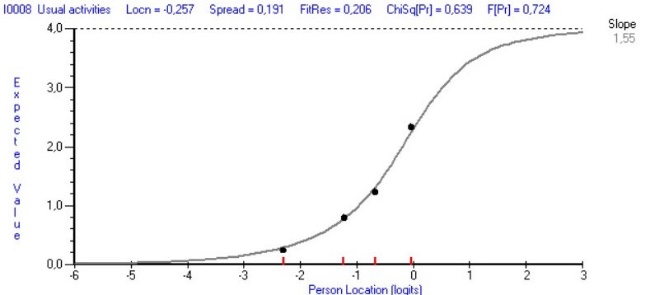** | |  |
| **22 Hobbies** | **23 Quality time** | |  |
| **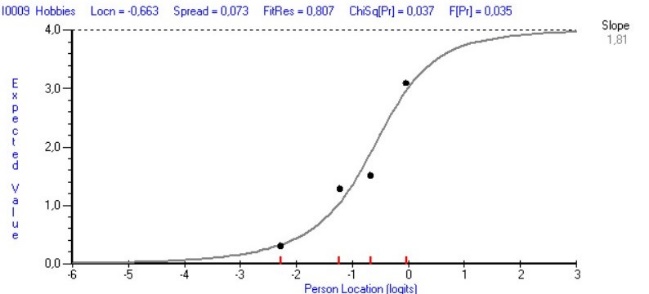** | **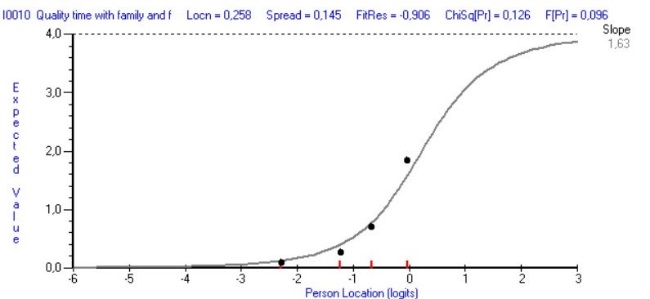** | |  |
| **24 Worry about sex life** | **25 Worry about infections** | |  |
| **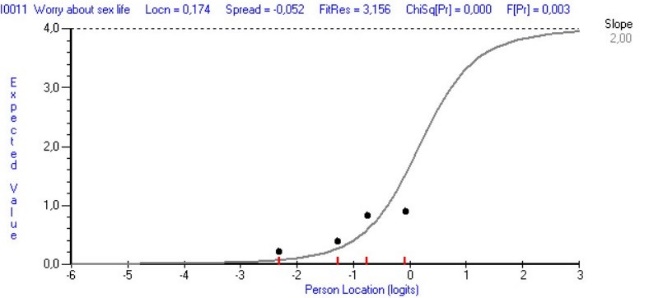** | **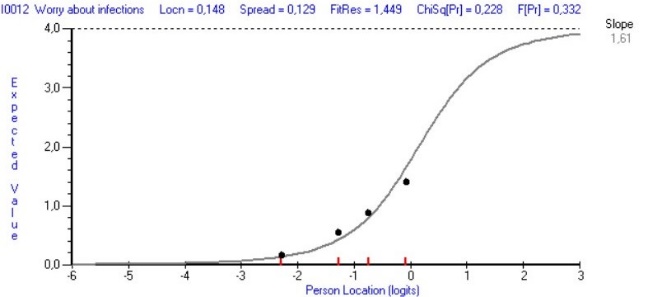** | |  |
| **26 Worry about physical appearance** | **27 Worry about finances** | |  |
| **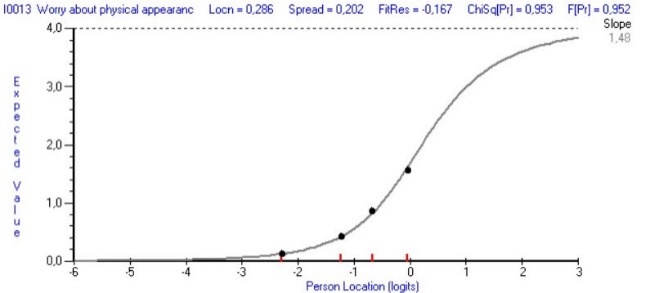** | **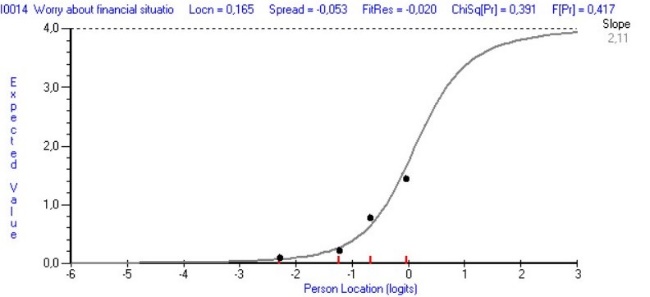** | |  |
| **28 Worry about illness worsening** | **29 Coping with illness/treatment** | |  |
| **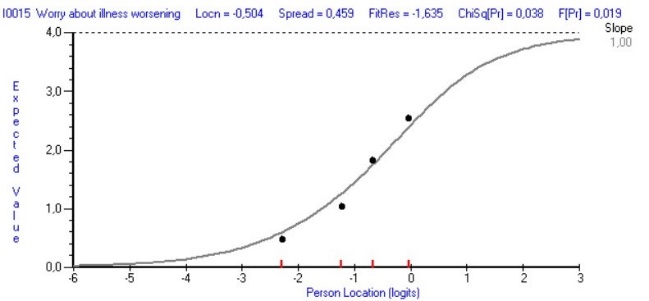** | **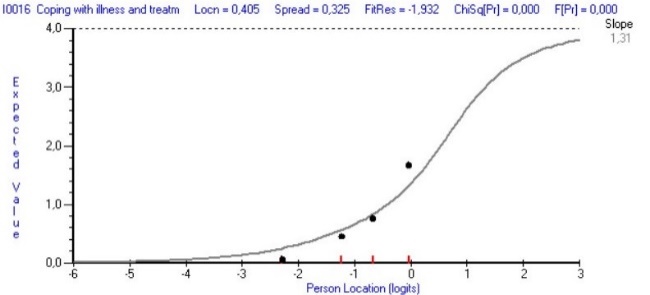** | |  |
| **33 Information about future** |  | |  |
| **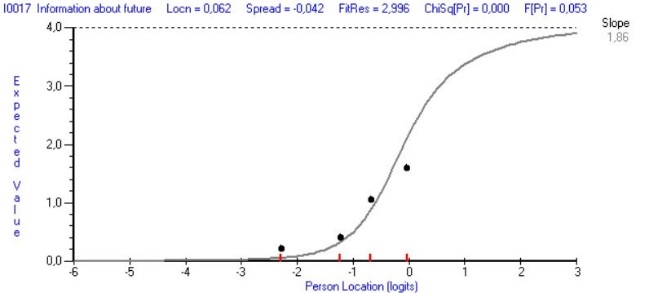** |  | |  |
|  |  | |  |
|  |  | |  |
|  |  | |  |
| **SUBSCALE HEALTHCARE SUPPORT** | | |  |
| **30 Advice from doctors/nurses** | **31 Knowledge of doctors/nurses** | |  |
| **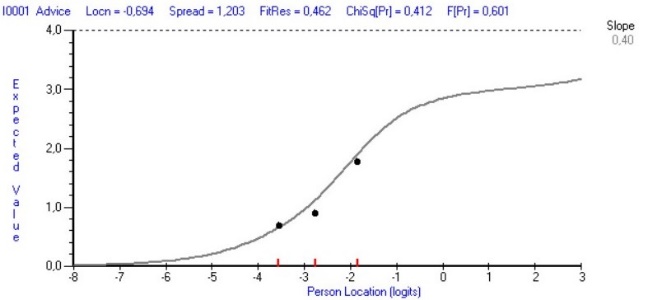** | **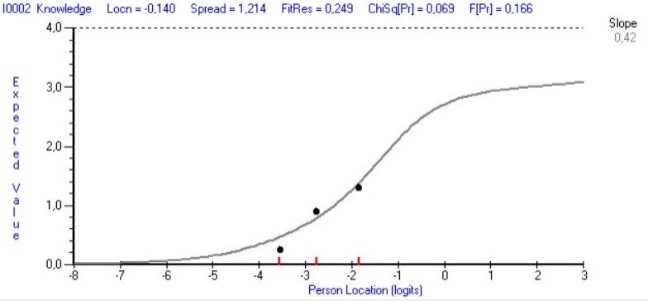** | |  |
| **32 Doctors/nurses show care/respect** |  | |  |
| **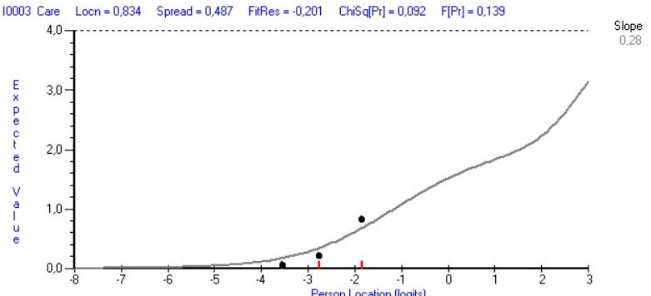** |  | |  |

**Supplemental Figure 4** Distribution (expressed in percent) of changes in scores on the total Myeloma Patient Outcome Scale for patients with multiple myeloma who report an important improvement (left-hand side) or an important deterioration (right-hand side) in their quality of life compared to those who reported no important change at time point 5. The ROC point indicates the ROC-based MID, the distribution-based MID indicates the 95% limit of error that was determined.

**
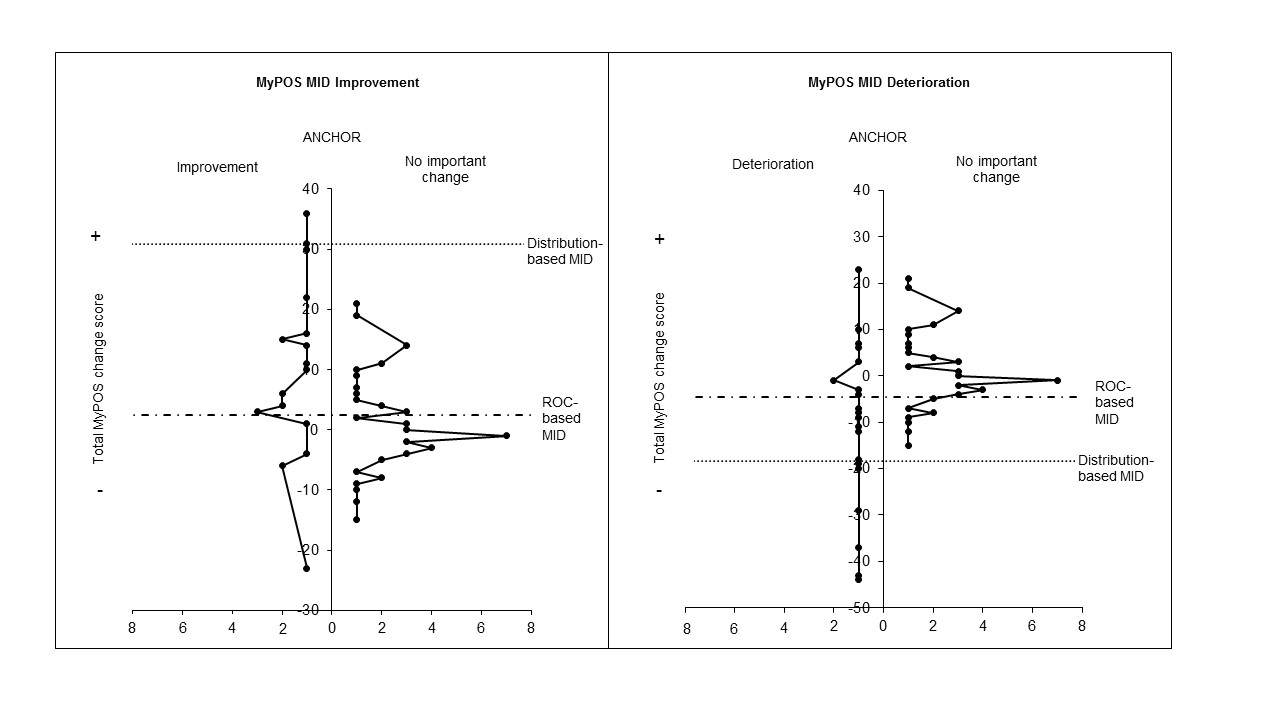
**
